# Impact of efflux in the development of multidrug resistance phenotypes in *Staphylococcus aureus*

**DOI:** 10.1186/s12866-015-0572-8

**Published:** 2015-10-24

**Authors:** Sofia Santos Costa, Miguel Viveiros, Adriana E. Rosato, José Melo-Cristino, Isabel Couto

**Affiliations:** Global Health and Tropical Medicine, GHTM, Unidade de Microbiologia Médica, Instituto de Higiene e Medicina Tropical, IHMT, Universidade Nova de Lisboa, UNL, Rua da Junqueira, 100, 1349-008 Lisbon, Portugal; Department of Pathology and Genomic Medicine, Center for Molecular and Translational Human Infectious Diseases Research, Houston Methodist Research Institute, Houston, TX USA; Centro Hospitalar Lisboa Norte E.P.E., Instituto de Microbiologia, Instituto de Medicina Molecular, Faculdade de Medicina, Universidade de Lisboa, Avenida Professor Egas Moniz, 1649-028 Lisbon, Portugal

**Keywords:** *Staphylococcus aureus*, Antimicrobials, Fluoroquinolones, Biocides, Multidrug resistance, Efflux

## Abstract

**Background:**

Efflux has been recognized as a resistance mechanism to antimicrobials in *Staphylococcus aureus*; however its role on the development of clinically relevant resistance is still poorly characterized. This study aimed to examine the impact of efflux on development of resistance to fluoroquinolones and other antimicrobials in *S. aureus* strains representing relevant phenotypes in terms of antibiotic susceptibility and efflux activity.

**Methods:**

Two closely related methicillin- and ciprofloxacin-resistant *Staphylococcus aureus* clinical strains, with different efflux capacity and the pan-susceptible strain ATCC25923 were exposed to constant concentrations of the efflux pump (EP) substrates ciprofloxacin, ethidium bromide and cetrimide. Parental and exposed strains were tested regarding their susceptibility towards antibiotics, biocides and ethidium bromide, efflux capacity and levels of EP gene expression. Occurrence of resistance-associated mutations was screened by sequencing.

**Results:**

Multidrug resistance phenotypes emerged upon exposure, independently of the substrate or its concentration, which were correlated with increased efflux capacity of the exposed strains. The temporal pattern of EP gene expression disclosed an early-response with high expression of several genes, followed by a late-response, characterized by overexpression of specific genes. The overall cell response was more pronounced for strains with an initial basal efflux activity. Remarkably, detection of the IS256 element in the promoter regions of *mgrA* and *norA*, in some cases associated with increased gene expression, suggests that these genes may be hot spots for IS256 insertion events.

The results obtained with exposure of ATCC25923 to ciprofloxacin were particularly striking, revealing a step-wise development of fluoroquinolone resistance, with a first efflux-mediated response, followed by the occurrence of a mutation in *grlA* that resulted in phenotypic resistance. Additionally, challenge by non-fluoroquinolone agents, particularly cetrimide, promoted cross resistance to fluoroquinolones, revealing the potential role of biocides as selective pressure for the emergence of resistance to these antibiotics.

**Conclusions:**

This study reveals efflux as a significant component of *S. aureus* resistance to fluoroquinolones and biocides and as a primary mechanism to withstand stress imposed by antimicrobials. This efflux-mediated response can result in the emergence of multidrug resistance in healthcare environments and should be taken into account in the management of this major pathogen.

**Electronic supplementary material:**

The online version of this article (doi:10.1186/s12866-015-0572-8) contains supplementary material, which is available to authorized users.

## Background

*S. aureus* is a frequent human colonizer and major pathogen. Besides its pathogenicity and virulence potential, the development and/or acquisition of resistance to antimicrobials (antibiotics and biocides) is of foremost importance, as the occurrence of strains with a multidrug resistance (MDR) phenotype is common. In particular, methicillin-resistant *S. aureus* (MRSA) strains have become a major problem in healthcare settings and in the community [1] as they are generally associated with increased burden regarding therapeutics and higher mortality rates than methicillin-susceptible *S. aureus* (MSSA) strains [2, 3].

Fluoroquinolones target the *S. aureus* topoisomerase IV (GrlA/B) and DNA gyrase (GyrA/B), inhibiting DNA replication [4]. Resistance to these antibiotics emerges swiftly and has been mainly attributed to the occurrence of spontaneous mutations in the quinolone resistance-determining region (QRDR) of the target genes *grlA*/*B* and *gyrA*/*B* [4]. In Europe, around 25 % of invasive *S. aureus* clinical isolates are resistant to fluoroquinolones, a rate that increases to almost 90 % among MRSA isolates [5]. Although fluoroquinolones are not used for the treatment of staphylococcal infections, their intensive use in the hospital [6] has been pointed out as a main selective factor for the emergence and dissemination of fluoroquinolone resistance in *S. aureus*, which, in turn, has been suggested to act as a selective advantage for MRSA strains in comparison with MSSA strains [7].

Resistance to fluoroquinolones may also arise by their extrusion via efflux pumps (EPs) [8], as reported in *S. aureus* clinical isolates [9–11] but considered clinically non-relevant [12]. Several multidrug EPs have been identified in *S. aureus*, including NorA, NorB, NorC, MepA and MdeA [13]. The few studies conducted to ascertain their contribution to fluoroquinolone resistance have associated their activity to a reduced susceptibility to this class of antibiotics [14–17]. Moreover, this same efflux activity could be linked to decreased susceptibility to additional antimicrobials such as biocides and dyes [14, 15, 18], highlighting the potential of these EPs to convey a MDR phenotype to *S. aureus* strains.

Recent studies provided additional data supporting the premise that efflux plays an important role in the emergence of resistance to antimicrobials in bacteria. Indeed, data from *Escherichia coli* and *Mycobacterium tuberculosis* demonstrate that efflux may be the cell’s first response to cope with these compounds, allowing them to endure their noxious effects until acquisition of a more stable resistance mechanism, such as mutation, that will then provide high-level resistance [19, 20]. Studies on *S. aureus* also demonstrated the role of efflux as a first-line defence mechanism towards noxious compounds [21, 22], a hypothesis that has been confirmed in clinical isolates [14–16, 23].

In this study, we aimed to highlight the relationship between efflux and mutation(s) throughout the process of emergence of resistance in *S. aureus*, by exposing a set of representative MSSA and MRSA strains to sub-inhibitory or inhibitory concentrations of antimicrobials that are known substrates of MDR EPs: the fluoroquinolone ciprofloxacin (CIP), the biocide cetrimide (CET) and the dye ethidium bromide (EtBr). The three strains studied included a fully susceptible reference MSSA strain and a pair of closely related clinical MRSA strains resistant to ciprofloxacin, which carry the same set of QRDR mutations but that differ in their efflux activity. The expression of genes coding for the main MDR EPs and their regulators was assessed at different time points of exposure and correlated with the resistance level towards fluoroquinolones and other antimicrobials and the temporal acquisition of mutations.

## Results

### Exposure of MSSA and MRSA strains to fluoroquinolones and biocides promotes a multidrug efflux response

The reference MSSA strain ATCC25923 and two clinical MRSA strains, SM2 and SM50, belonging to ST2246-t037 (clonal complex 8), were each subjected to six different exposure regimens for a period of 20-days: exposure to the minimum inhibitory concentration (MIC) and to half MIC of EtBr, CIP and CET (Additional file 1: Figure S1). Bacterial growth was observed in the presence of the MIC of each compound during day 1 of most exposure regimens, albeit at different growth rates (data not shown), probably due to: a) the different growth conditions used in comparison to the MIC determination protocol and b) the initial gradual physiological adaptation and survival of bacteria to the MIC of the antimicrobial to which they were exposed.

Occurrence of contaminations was ruled out by PFGE analysis of *Sma*I macrorestriction profiles of the strains before (P0) and after (P20) each exposure experiment (Additional file 1: Figure S2).

**Fig. 1:**
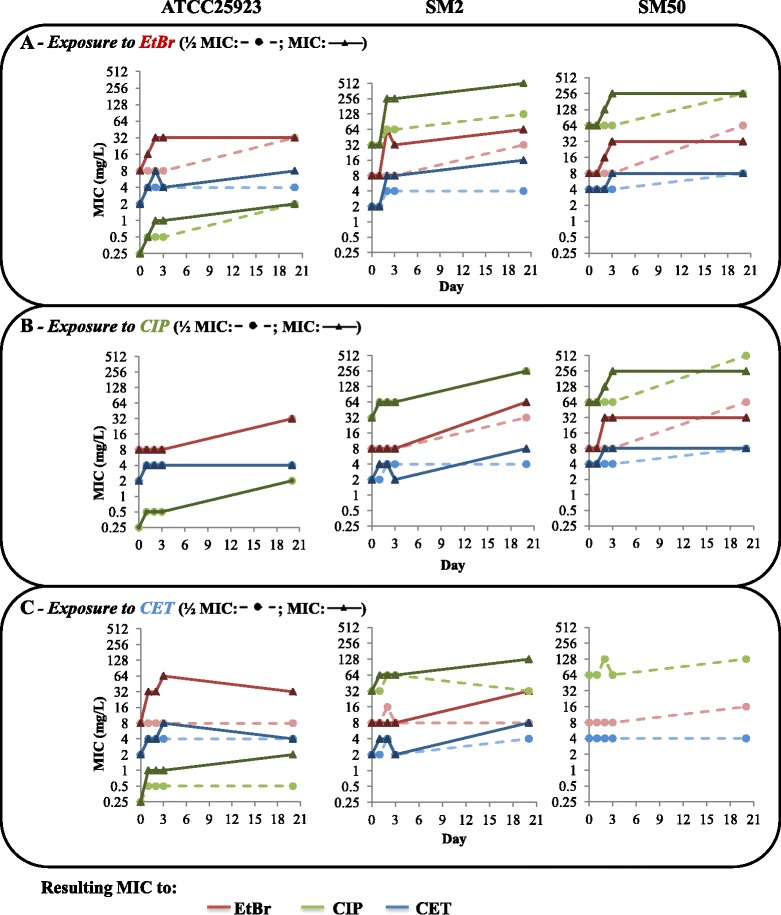
Evolution of MIC values (mg/L) of the EP substrates for the strains in study. The data presented correspond to the MICs of ethidium bromide (EtBr, red), ciprofloxacin (CIP, green) and cetrimide (CET, blue) throughout the 20-day exposure to EtBr (**a**), CIP (**b**) and CET (**c**) at half MIC (dotted lines) or at the MIC (full lines) (data available for days 0, 1, 2, 3 and 20^th^). No growth was obtained for strain SM50 at the CET concentration corresponding to the MIC

Changes in the susceptibility profiles of the strains were monitored by determination of MICs of the EP substrates during exposure (Fig. 1, Additional file 1: Tables S1-S3). We observed an overall MIC increase from two-fold to 16-fold, although exposure to the MIC did not always result in the highest levels of resistance (e.g., ATCC25923 or SM50 exposed to CIP, Fig. 1b). EtBr and CIP promoted higher MIC increases (mainly four- to 16-fold) than CET (mostly two- to four-fold). The strains responsiveness to the EP substrates differed according to their initial efflux activity [14]; strains SM2 and ATCC25923 (with basal efflux activity), showed higher MIC increases than strain SM50 (higher initial efflux activity), which was the least responsive strain. Moreover, absence of growth was observed for strain SM50 exposed to the cetrimide MIC (Fig 1c, Additional file 1: Table S3).

The data shown in Fig. 1 revealed that, in most cases, the MIC increases occurred in the first days of exposure, and in some conditions, more swiftly in the presence of the MIC of the EP substrate (e.g., ATCC25923 exposed to EtBr, Fig 1a). Strikingly, the three strains achieved similar final MICs of EtBr and CET (32–64 mg/L and 4–8 mg/L, respectively) independently of the exposure condition (EP substrate or its concentration). Of particular interest, exposure of strain ATCC25923 to each EP substrate determined an increase in the MIC values of fluoroquinolones, namely from 0.25 to 2 mg/L for CIP; from 0.5 to ≥ 8 mg/L for norfloxacin and from 0.25 to 0.5-1 mg/L for levofloxacin, with the exception of exposure to CET at half MIC (Additional file 1: Table S1). In contrast, no significant alterations in susceptibility level were found for the other antibiotics tested: oxacillin; penicillin; vancomycin; chloramphenicol and tetracycline.

The exposure experiments also resulted in increased resistance to the quaternary ammonium compounds cetylpyridinium chloride, benzalkonium chloride and dequalinium chloride; to tetraphenylphosphonium bromide; to the diamidine pentamidine and in a lesser extent to the bisbiguanidine chlorhexidine (Additional file 1: Tables S1-S3).

The next set of experiments confirmed that the MDR phenotypes observed were associated with increased efflux, as assessed by MIC determinations in the presence of the efflux inhibitors thioridazine (TZ) (Table 1) and verapamil (VER) (Additional file 1: Table S4). It has been suggested that a four-fold MIC reduction in the presence of an efflux inhibitor indicates inhibition of increased efflux activity [14, 18, 24]. In this work, we observed that TZ and VER were able to reduce the increased MICs of EtBr, CIP and CET by two to 32-fold (Table 1, Additional file 1: Table S4). This inhibitory effect was more pronounced for strains ATCC25923 and SM2 exposed to EtBr.

**Table 1:** Effect of TZ on EP substrates MICs before and after the exposure processes

MIC (mg/L) after exposure to:	ᅟ	ᅟ	ᅟ	ᅟ	ᅟ	ᅟ	ᅟ	ᅟ	ᅟ	ᅟ	ᅟ	ᅟ	ᅟ	ᅟ
			EtBr				CIP				CET			
	Original MIC (mg/L)		½ MIC		MIC		½ MIC		MIC		½ MIC		MIC	
	No EI	+ TZ	No EI	+ TZ	No EI	+ TZ	No EI	+ TZ	No EI	+ TZ	No EI	+ TZ	No EI	+ TZ
***ATCC25923***														
**EtBr**	8	**1 (↓8x)**	32	**4 (↓8x)**	32	**1 (↓32x)**	32	**8 (↓4x)**	32	**4 (↓8x)**	8	**2 (↓4x)**	32	**4 (↓8x)**
**CIP**	0.25	0.125 (↓2x)	2	**0.25 (↓8x)**	2	**0.25 (↓8x)**	2	**0.5 (↓4x)**	2	**0.5 (↓4x)**	0.5	0.25 (↓2x)	2	**0.5 (↓4x)**
**CET**	2	**0.25 (↓8x)**	4	**0.5 (↓8x)**	8	**0.125 (↓64x)**	4	**0.5 (↓8x)**	4	**0.5 (↓8x)**	4	**0.5 (↓8x)**	4	**0.5 (↓8x)**
***SM2***														
**EtBr**	8	**2 (↓4x)**	32	**1 (↓32x)**	64	**2 (↓32x)**	32	**4 (↓8x)**	32	**2 (↓16x)**	8	**1 (↓8x)**	32	**4 (↓8x)**
**CIP**	32	16 (↓2x)	128	**16 (↓8x)**	512	**32 (↓16x)**	256	**32 (↓8x)**	256	**16 (↓16x)**	32	**8 (↓4x)**	128	**32 (↓4x)**
**CET**	2	**0.5 (↓4x)**	4	**0.25 (↓16x)**	16	**4 (↓4x)**	4	2 (↓2x)	8	**1 (↓8x)**	4	**0.5 (↓8x)**	8	**2 (↓4x)**
***SM50***														
**EtBr**	8	**1 (↓8x)**	64	**8 (↓8x)**	32	**8 (↓4x)**	64	**4 (↓16x)**	32	**4 (↓8x)**	16	**2 (↓8x)**	---	---
**CIP**	64	**16 (↓4x)**	256	**64 (↓4x)**	256	**64 (↓4x)**	512	**64 (↓8x)**	256	**64 (↓4x)**	128	**32 (↓4x)**	---	---
**CET**	4	**0.5 (↓8x)**	8	**1 (↓8x)**	8	**1 (↓8x)**	8	**2 (↓4x)**	8	**2 (↓4x)**	4	**1 (↓4x)**	---	---

The values in brackets correspond to the decrease (↓ n-fold) of the MICs in the presence of a sub-inhibitory concentration of TZ relatively to the original values (absence of efflux inhibitor); decreases of  ≥ 4-fold are highlighted by bold-type letters

**Fig. 2:**
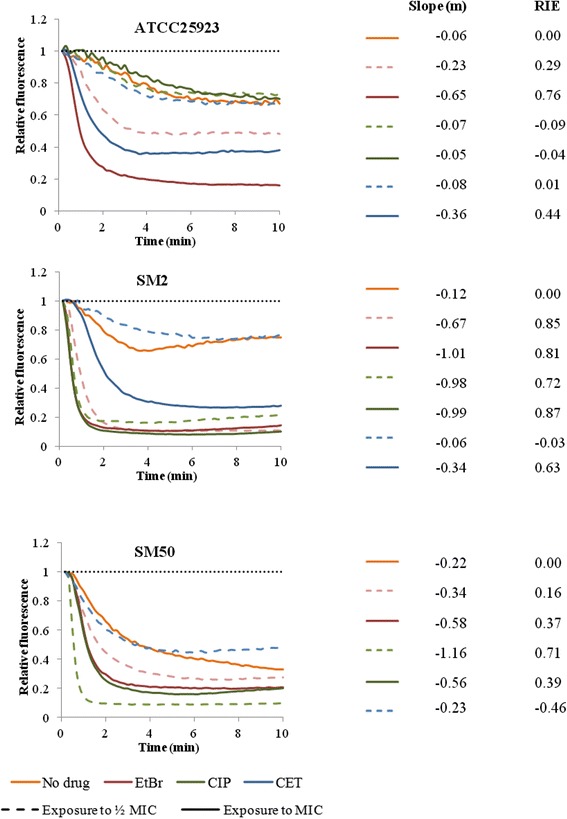
Assessment of EtBr efflux for the strains in study. Efflux capacity was evaluated at the beginning (orange) and at the end of the exposure process to ethidium bromide (EtBr, red), ciprofloxacin (CIP, green) and cetrimide (CET, blue) at half MIC (dashed lines) and at the MIC (full lines). All assays were conducted in the presence of 0.4 % glucose. EtBr-loaded cells were obtained by incubation with 200 mg/L of VER plus the following EtBr concentrations: 0.125 mg/L (ATCC25923: P0, CIP(½MIC)_P20, CIP(MIC)_P20, CET(½MIC)_ P20; SM2: P0, CET(½MIC)_P20; SM50_CET(½MIC)_P20); 0.25 mg/L (ATCC25923: EtBr(½MIC)_P20, CET(MIC)_P20; SM2: CET(½MIC)_P20, CET(MIC)_P20; SM50: P0, EtBr(½MIC)_P20, EtBr(MIC)_P20, CIP(MIC)_P20); 0.5 mg/L (ATCC25923_EtBr(MIC)_P20; SM2: EtBr(½MIC)_P20, EtBr(MIC)_P20, CIP(MIC)_P20; SM50_CIP(½MIC)_P20). The data presented was normalized against the data obtained in conditions of no efflux (dotted line, cells incubated without glucose in the presence of 200 mg/L of VER). The slope (m) of the EtBr efflux curves was calculated by a linear regression of the values obtained in the first minutes of the assay and it relates to the rate of EtBr efflux in each condition tested. The Relative Index of Efflux activity (RIE) values were calculated as described in Methods and allow the direct comparison of the EtBr efflux activity of the strain after exposure (P20) to their initial efflux activity (P0)

The efflux activity of each exposed strain was further evaluated by real-time fluorometry (Fig. 2). The slope of each EtBr efflux curve was determined and correlated to the rate of efflux, as well as the Relative Index of Efflux activity (RIE), which corresponds to the exposed cells overall capacity to efflux EtBr relatively to their original status. The initial efflux activity of each strain is indicated in orange in Fig. 2 and confirms that the initial basal and naive efflux activity of both ATCC25923 and SM2 was lower than that of SM50 (lower slope value). Most of the conditions tested resulted in increased efflux activity, with the exception of strains exposed to the sub-inhibitory concentrations of CET and ATCC25923 exposed to CIP. Also, in most cases, higher EtBr efflux (higher RIE values and lower slope values) was detected with the highest concentrations of substrate (Fig. 2).

Altogether, these data revealed that increased antimicrobial resistance in all the exposed strains correlates with an augmented efflux capacity induced by the contact with substrates of multidrug EPs.

### Evaluation of EP gene expression levels

**Fig. 3:**
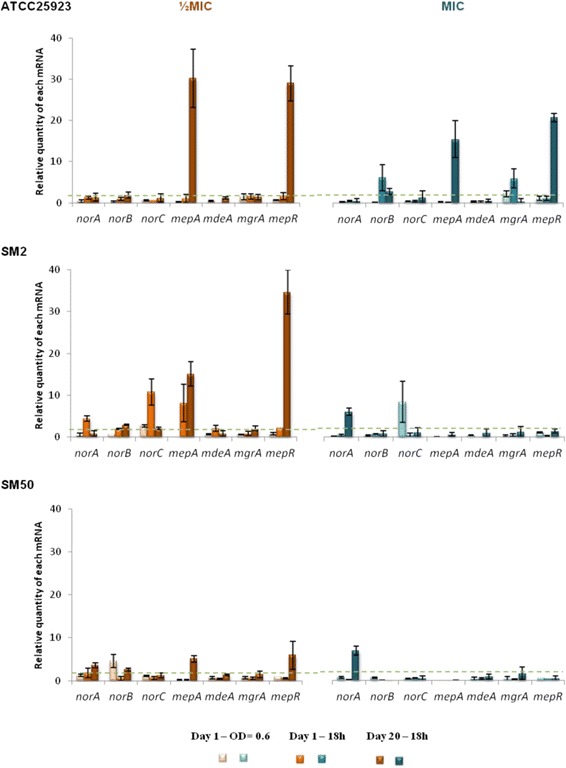
Expression levels of EP and regulator genes of the strains in study during exposure to EtBr. Gene expression was measured in the presence of EtBr at half MIC (orange) and at the MIC (blue) relatively to the drug-free growth condition. The results are presented as the mean and standard deviation of at least two independent assays performed with total RNA. Overexpression was considered for values superior to 2 (cut-off value represented by the green dashed line)

**Fig. 4:**
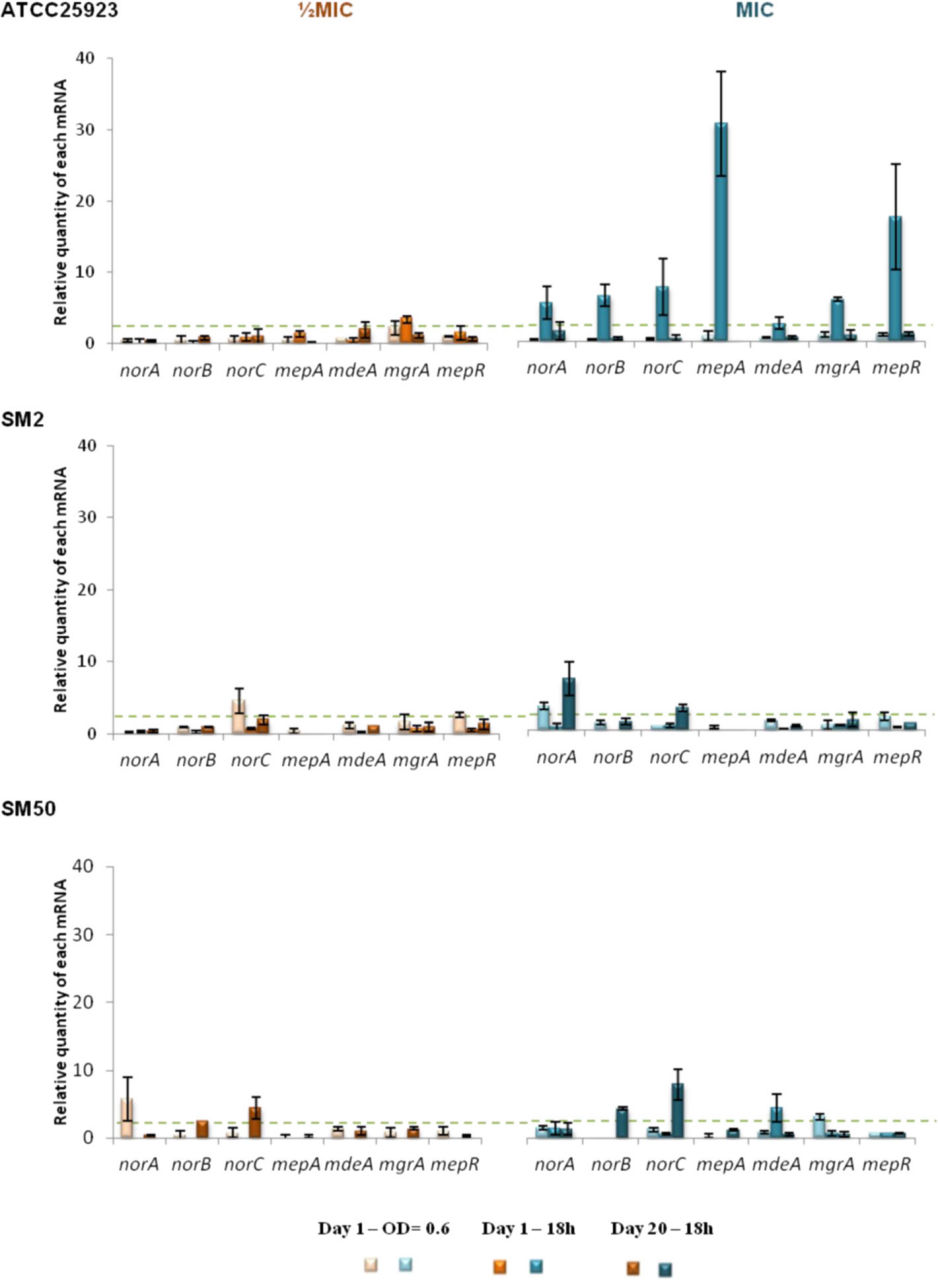
Expression levels of EP and regulator genes of the strains in study during exposure to CIP. Gene expression was measured in the presence of CIP at half MIC (orange) and at the MIC (blue) relatively to the drug-free growth condition. The results are presented as the mean and standard deviation of at least two independent assays performed with extracted total RNA. Overexpression was considered for values superior to 2 (cut-off value represented by the green dashed line)

**Fig. 5:**
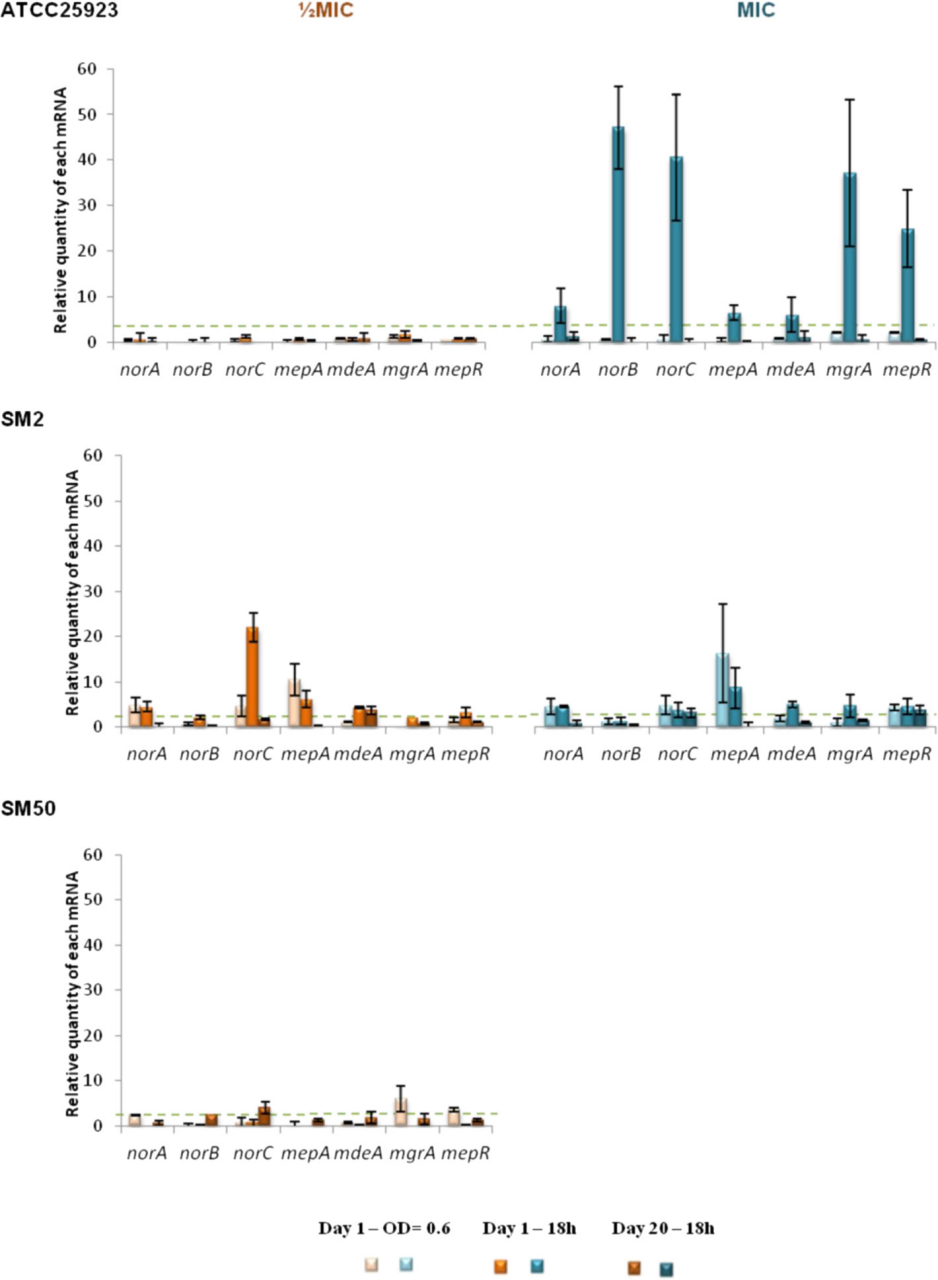
Expression levels of EP and regulator genes of the strains in study during exposure to CET. Gene expression was measured in the presence of CET at half MIC (orange) and at the MIC (blue) relatively to the drug-free growth condition. The results are presented as the mean and standard deviation of at least two independent assays performed with extracted total RNA. Overexpression was considered for values superior to 2 (cut-off value represented by the green dashed line)

To ascertain which EPs were responsible for the induced MDR phenotypes, we measured the expression levels of the *S. aureus* MDR EP genes *norA*, *norB*, *norC*, *mdeA* and *mepA*, as well as their regulatory genes *mgrA* and *mepR*, at different stages of exposure. Changes in gene expression were considered an early response when occurring during day 1 of exposure, and a late response when detected at day 20 (Fig. 3–5, Additional file 1: Tables S5-S7).

#### Exposure to EtBr

Figure 3 and Additional file 1: Table S5 illustrate a similar genetic response for all strains to EtBr, consisting of an early response with low overexpression levels of *nor* genes (*norB* in ATCC25923 and SM50, and *norA*/*B*/*C* in SM2), and a late response with reduction of expression levels and/or a shift in the genes overexpressed (predominant high expression of *mepA* and its regulator *mepR*). This late and specific response via *mepA*/*mepR* genes was consistently observed at half MIC of EtBr for the three strains. In contrast, at the MIC, only ATCC25923 showed overexpression of *norB* and *mepA*/*mepR* whereas in strains SM2 and SM50 only increased expression of *norA* was detected.

#### Exposure to ciprofloxacin

A different genetic response was detected under exposure to CIP (Fig. 4, Additional file 1: Table S6). Compared to EtBr exposure, lower levels of gene expression were found at either CIP concentration tested. An exception was strain ATCC25923, which presented an early response to the CIP MIC consisting of increased expression of all genes, in particular *mepA*/*mepR* (Fig. 4, Additional file 1: Table S6). In contrast, the two CIP-resistant strains SM2 and SM50 showed a predominance of *nor* genes overexpression in both early and late responses, which varied with the concentration and over time (Fig. 4, Additional file 1: Table S6).

#### Exposure to cetrimide

Exposure to CET also produced different patterns of gene expression (Fig. 5, Additional file 1: Table S7). Strain ATCC25923 presented a strong early response when exposed to the MIC, with high levels of expression for all the genes tested, while no changes in expression were seen on exposure to half MIC. The two clinical strains had very different expression patterns. SM2 revealed a strong early response, with increased expression of nearly all genes tested followed by a weak late response, mediated by *mdeA* or *norC*, depending on the concentration used. Conversely, SM50 showed only a low level overexpression of the *nor* genes either in the early (*norA*) or late (*norB*/*C*) response.

### Screening for the emergence of mutations associated with resistance to fluoroquinolones

**Fig. 6:**
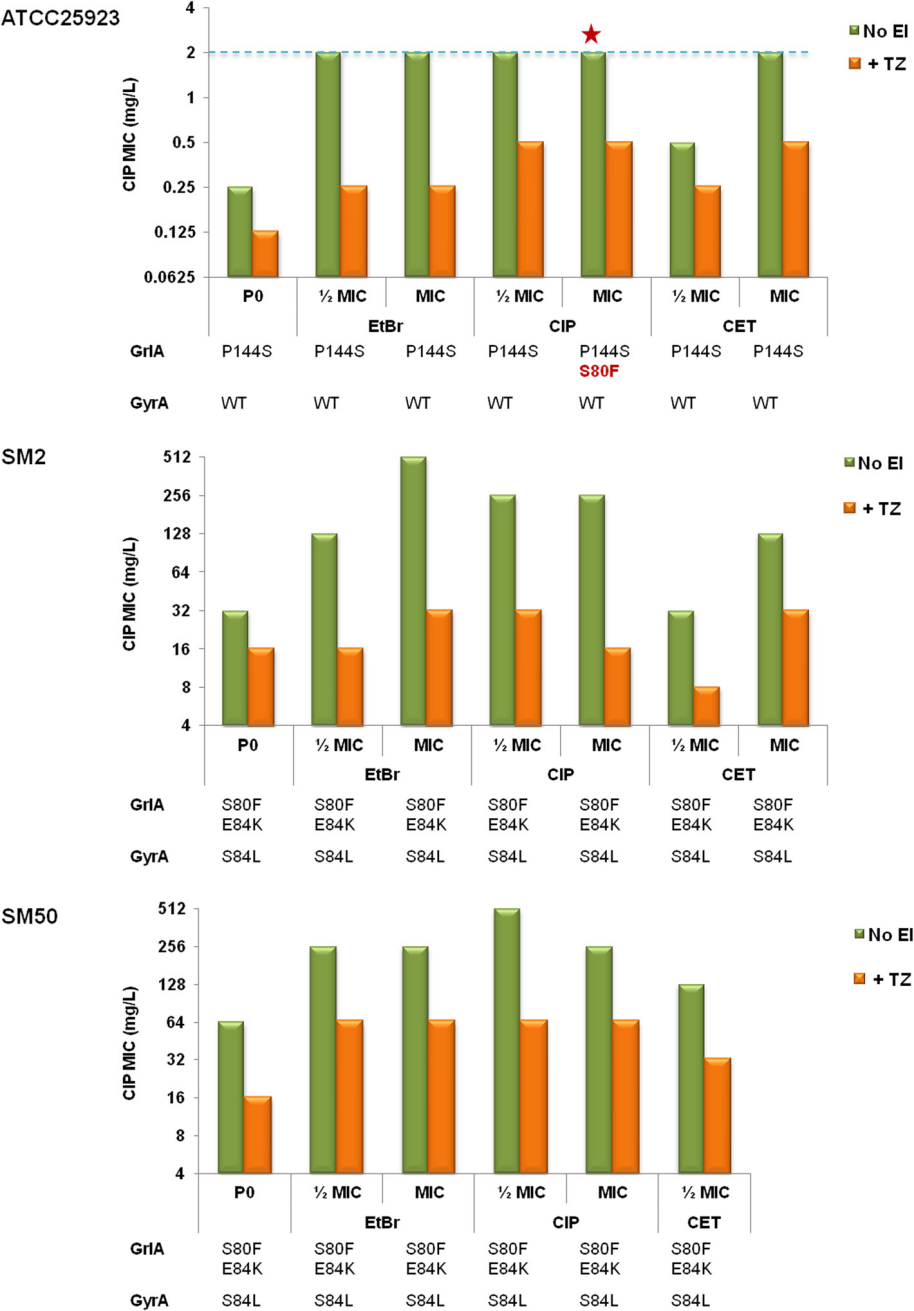
Effect of thioridazine on the ciprofloxacin MIC for the strains exposed to EP substrates. MIC values were determined for each strain prior to exposure (P0) and after each exposure process (P20) in the absence (green columns) and in the presence (orange columns) of the efflux inhibitor thioridazine (TZ) at the sub-inhibitory concentration of 12.5 mg/L. The strain ATCC25923 exposed to the ciprofloxacin MIC was the single one in which a fluoroquinolone-resistance mutation (S80F in the QRDR region of GrlA) occurred during an exposure process (red star). The breakpoint for considering intermediate resistance to ciprofloxacin (according to CLSI guidelines) is represented by a blue dashed line

The occurrence of mutations associated with resistance to fluoroquinolones was screened by analysis of the QRDR of *grlA* and *gyrA* genes for the strains exposed to ciprofloxacin. The CIP-susceptible strain ATCC25923 (MIC of 0.25 mg/L) contains a wild-type GyrA but carries a mutation in GrlA, P144S, which has been reported for some *S. aureus* isolates with a CIP MIC of 0.25 mg/L [17, 25], thus not associated with resistance. This strain did not gain mutations throughout exposure to half MIC of ciprofloxacin. However, exposure to the ciprofloxacin MIC resulted in an increased CIP MIC of 2 mg/L (Figs. 1 and 6, Table 1) and the acquisition of the mutation S80F in GrlA, while GyrA remained unchanged. Screening of the *grlA* QRDR of this strain in intermediate stages of the exposure process revealed that this mutation occurred at day 5. This mutation is one of the most commonly ascribed to GrlA in *S. aureus* clinical isolates and has been associated with ciprofloxacin resistance levels that vary between 2 and 12.5 mg/L [25, 26], corresponding to resistance according to CLSI and EUCAST guidelines [27, 28].

Importantly, ATCC25923 strain also revealed a final CIP MIC of 2 mg/L following exposure to EtBr and CET (at the MIC) but without any detectable occurrence of mutations in the QRDR of *grlA*/*gyrA* genes, indicating that in these exposure conditions, the increased fluoroquinolone resistance was solely due to augmented efflux.

The CIP-resistant strains SM50 and SM2 already carried by the time of their isolation a double mutation in GrlA (S80F/E84K) accompanied by a single mutation in GyrA (S84L), a combination associated with high level resistance to ciprofloxacin (MICs ranging from 100 to > 800 mg/L) [26]. The QRDR region of their *grlA*/*gyrA* genes was also sequenced after CIP exposure, but no additional mutations were detected, indicating that the observed increased resistance to fluoroquinolones (Figs. 1 and 6, Additional file 1: Tables S1-S3) is solely attributable to increased efflux activity, as confirmed by the results with efflux inhibitors (Fig. 6, Table1 , Additional file 1: Table S4). Screening for mutations in the strains exposed to EtBr and CET also showed that no mutations occurred during those exposure processes.

### Screening for the emergence of mutations in *norA* and *mgrA* promoters

**Fig. 7:**
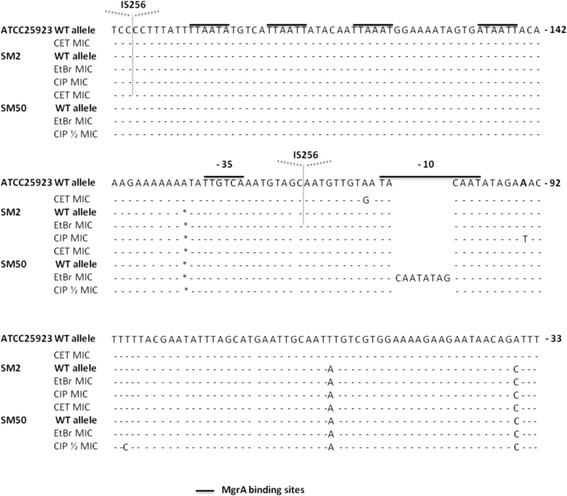
Location of the *norA* promoter mutations found in *norA* overexpressing strains following antimicrobial exposure. The nucleotides are numbered taking into consideration the *norA* starting codon. MgrA binding sites are identified by black lines. The consensus motifs −35 and −10 are also identified in black lines. The symbol * identifies the nucleotide −132 (A) which is absent in SM2 and SM50 *norA* promoter sequence. The sites of IS256 insertion within the *norA* promoter region of strain SM2 exposed to CET or EtBr MIC are also indicated (dotted arrows)

The occurrence of mutations in the *norA* promoter region has been frequently associated with *norA* overexpression [15, 23]. Therefore, we screened for mutations in *norA* promoter for exposed strains showing *norA* overexpression either in an early- or late-response. Different mutations were encountered (Fig. 7), all already described by other authors [23, 29, 30], which included the transitions A → G and T → C and the transversion A → T, at positions −107, −89 and −94 (the nucleotide assigned to the transcription initiation site) relatively to translation start. A duplication event resulting in the insertion of the sequence CAATATAG in the −10 consensus motif was also encountered. Of these mutations, only the transversion A → T (−94) in strain SM2 exposed to CIP MIC and the duplication event for strain SM50 exposed to the EtBr MIC were associated with *norA* overexpression at the 20^th^ day of exposure.

An unexpected result was the independent detection of the insertion element IS256 in the *norA* promoter region of strain SM2 upon exposure to the MIC of EtBr, between the consensus motifs −35 and −10, and to the MIC of CET, upstream of the promoter region and MgrA binding sites, with a probable association with increased *norA* expression only in the former. Rapid screening revealed that no IS256 insertion had taken place in the promoter regions of *norB* and *norC* in strain SM2, despite detection of increased expression of these two genes (data not shown).

No mutations were identified in the promoter region of *mgrA* for *mgrA* overexpressing strains, although a copy of IS256 was detected upstream of the *mgrA* putative promoter region in unexposed SM2 and SM50 strains, which may have affected *mgrA* expression and consequently, of genes regulated by MgrA, namely *norA/B/C*.

Despite the observed overexpression of *mepA/mepR* of strains ATCC25923 and SM2 exposed to EtBr, no mutations were found in the promoter region of the *mepRA* operon (data not shown). However, the mutation E32K was detected in the regulator protein MepR for strain SM2 exposed to EtBr, which may affect MepR function.

## Discussion

Antimicrobial resistance is one of the most critical challenges of our time. Several studies have revealed that resistance mediated by efflux is an important component of antimicrobial resistance in *S. aureus* clinical isolates [14–17]. The purpose of the present study was to disclose the impact of efflux on emergence of antimicrobial resistance, by exploring the effect of different antimicrobials at different concentrations and times of exposure in a group of representative strains, which included both MSSA and MRSA strains. Our aim was to provide a better understanding of the contribution of each multidrug EP to resistance and to gain further insights in the process of development of efflux-mediated resistance to antimicrobials in *S. aureus* and its relationship with mutation-based resistance. The study was restricted to three representative, well characterized strains, in order to allow a comprehensive interpretation of the patterns obtained. The strains selected were *S. aureus* ATCC25923, a fully susceptible reference strain, and two MRSA clinical strains that belong to a nosocomial clone that has been detected in this major Lisbon hospital [31], ST2246-t037 (CC8), differing in their initial efflux activity [14, 18].

The results obtained clearly suggest that long-term exposure of MSSA (ATCC25923) and MRSA (SM2 and SM50) strains to the selected EP substrates resulted in the development of a MDR phenotype, with increased resistance to these agents as well as cross-resistance to other antimicrobials (including norfloxacin, several quaternary ammonium compounds and pentamidine). The MDR phenotype emerged independently of the EP substrate, its concentration and the strain’s initial susceptibility phenotype or efflux capacity.

The emergence of an efflux-mediated MDR phenotype upon exposure to antimicrobials has also been observed in previous studies [23, 24], showing that exposure to a substrate of a multidrug EP promotes cross-resistance to other substrates of the same EP. This behavior towards selective pressure was also observed by Furi *et al*. [30]. One of the most important findings of this work was that the challenge of a susceptible strain, ATCC25923, by constant sub-inhibitory or inhibitory concentrations of non-fluoroquinolone agents, particularly cetrimide, promoted cross-resistance to fluoroquinolones, revealing the potential role of these agents as a selective pressure for the emergence of resistance to fluoroquinolones in healthcare environments.

The genetic responsiveness of the strains to the different EP substrates suggests a temporal differentiation in the activation of MDR EP genes (cf. Fig. 3–5, Additional file 1: Tables S5-S7). First, an early-response, occurring throughout day 1 of exposure, where the strains responded in a strong and non-specific manner by increasing the expression of several EP genes. This was followed by a more specific late-response, which occurred following prolonged exposure to the stimulus, in which the number of EP genes overexpressed and/or their level of expression decreased. This reduction in expression levels may occur because the number of EP proteins in the cell membrane required to cope with the antimicrobials may have reached a maximum value, suggesting that the reduced expression level observed at day 20 may be the necessary to compensate for protein turnover.

In a closer look, different patterns of gene expression were found. Some genes were predominantly overexpressed upon exposure to EtBr and CIP (*mepA* and the *nor* genes, respectively), in accordance with the substrate profile described for these MDR EPs [13]. Regarding CET, a preferable expression pattern of EP genes could not be established since a strong response with several genes was observed. This non-specific response may reflect the broader effect that this membrane-active compound exerts upon the bacterial cell [32], which triggers a global stress response. A correlation between the substrate concentration and increased gene expression was only observed for strain ATCC25923, which was independent of the substrate. For the clinical strains SM2 and SM50, increasing concentrations of EP substrates did not result in a stronger nor earlier response.

Although the results obtained for these three strains cannot be over generalized, they clearly show an overall pattern of *S. aureus* responsiveness upon challenge with antimicrobials, which is efflux driven and build upon each strain characteristics and may assist the design of future studies on antimicrobial resistance prevention. This overall response was less pronounced for SM50, the only strain which presented an initial well-established increased efflux activity. This difference translated in the distinct behaviour of both clinical strains in response to EtBr and CET. While strain SM2 presented a strong early response with overexpression and de novo expression of several EP genes, the response of SM50 was comparably weaker (cf. Fig. 3–5). This dissimilar behavior is particularly striking as these are very closely related strains, as demonstrated by PFGE analysis and illustrates the multiple efflux-mediated responses that *S. aureus* can display within the same lineage, a finding in contrast to results from other studies [33]. Regarding the response to CIP, both strains (already carrying QRDR mutations conferring high-level resistance) showed mild values of EP gene overexpression, which may account for the additional level of resistance attributed to efflux. Previous data suggested that clinical strains are primed to efflux noxious compounds, a trait that may be attributable to their prior exposure to antimicrobials in the hospital environment [14]. The susceptible reference strain ATCC25923, with an initial basal efflux activity, presented a behavior similar to SM2, with strong responses to all three substrates. This is a prototype strain with no prior exposure to antimicrobials and not primed for efflux, thus requiring higher levels of EP gene expression to cope with the noxious agents. In a previous work, exposure of ATCC25923 to EtBr triggered a preferred induction of the *norA* gene [24], whereas in the present study a MepA-mediated response to EtBr was noted. This difference could be due to the different exposure methodology used (exposure to constant versus step-wise, increasing EtBr concentrations). These data support previous findings suggesting that the same strain may respond to the same substrate via different efflux systems, depending on the concentration of the substrate and time of contact [14, 23].

Screening for mutations in *norA* promoter region revealed that stable *norA* overexpression was correlated with the occurrence of mutations in its promoter region. Most striking, we could also observe the insertion of the IS256 element in the *norA* and *mgrA* promoter regions, although not always associated with increased gene expression. Multiple IS256 transpositions have been observed in the genome of *S. aureus* and correlated with reduced susceptibility to vancomycin, as a copy of IS256 was found in the promoter region of genes associated with glycopeptide resistance originating hybrid promoters, probably responsible for an increased gene expression [34]. Other studies have also shown that exposure to antibiotics such as CIP, vancomycin, chloramphenicol, linezolid and spectinomycin activates spontaneous transposition of IS256 which could be associated with a decrease of antibiotic susceptibility [35–37]. Moreover, it has been shown that IS256 insertion in the promoter regions of the genes *mecA* in *Staphylococcus sciuri* [38] and *llm* in *S. aureus* [39] is involved in higher transcription rates of both genes with a concomitant increased resistance to methicillin. In this study, we show that exposure to non-antibiotics may also activate spontaneous transposition of IS256 and that *norA* and *mgrA* promoters may be hot spots for IS256 insertion , an event that, to our knowledge, is here described for the first time, further supporting the important role of efflux on the global *S. aureus* stress response.

The evolution of the susceptibility profile and gene expression analysis upon exposure demonstrated that efflux is a first-line response to antimicrobials. For the two clinical strains, it was demonstrated that efflux is an important component of resistance as these ciprofloxacin-resistant strains, harboring resistance mutations prior to exposure, became even more resistant to these antibiotics, not by acquiring additional mutations but by increasing their efflux activity. This observation was empathized by the inclusion of a fully susceptible reference strain for which the acquisition of fluoroquinolone resistance mutations follows the activation of EPs, which confer a low but clinically significant level of resistance that allows survival of bacteria even under MIC exposure. Significantly, this same resistance phenotype could be achieved by exposure to non-fluoroquinolone antimicrobials. These findings provide further evidence that efflux is an important player in the emergence of fluoroquinolone resistance in *S. aureus*.

## Conclusions

The last decade witnessed a growing awareness of the clinical importance of efflux as a mechanism of resistance to antimicrobial compounds. In recent years, a new perspective on the role played by MDR efflux systems has been emphasized, which are now perceived as important players in the emergence of resistance in bacteria [40]. In addition, a wide number of toxic compounds have been identified as substrates of these systems [41]. The findings of this study show that MDR EP substrates, including fluoroquinolones and biocides, compounds that are commonly used in hospital environments, promote a physiological response by *S. aureus,* which is based on efflux and persists over time with the maintenance of the stimulus. In the case of the pan-susceptible ATCC25923 strain, this efflux-mediated response resulted in detectable resistance to fluoroquinolones, in a step-wise process; a first step with activation of multidrug EP genes followed by a second step with acquisition of QRDR mutations. These are promising results, as they establish the key role of efflux in the first-line response to these antimicrobial agents until more stable and efficient forms of resistance appear*.* Moreover, they suggest that exposure to non-fluoroquinolone agents may act as selective pressure for the maintenance and dissemination of fluoroquinolone resistance, reportedly a selective factor for the persistence of MRSA strains in the hospital [7].

## Methods

### Bacterial strains

*S. aureus* ATCC25923 is a fully susceptible reference strain used as control in susceptibility testing while SM50 and SM2 are representative MRSA strains, from a collection of 52 ciprofloxacin-resistant *S. aureus* clinical isolates, originating from a 1, 300-bed teaching hospital in Lisbon, Portugal [14]. The SM50 and SM2 strains are genetically related, differing in their macrorestriction PFGE patterns in the size of a single *Sma*I-fragment of ca. 400 kb; in addition these strains carry the same set of QRDR mutations in GrlA (S80F/E84K) and GyrA (S84L) [14, 18]. Despite these similarities, SM50 shows a higher fluoroquinolone resistance level, previously correlated with an increased efflux activity, which was also associated with increased resistance to biocides and EtBr. In comparison, strains SM2 and ATCC25923 have been previously characterized as presenting only basal efflux activity [14, 18].

#### Antimicrobials and efflux inhibitors

The compounds were acquired from different sources, as follows: CIP (ciprofloxacin), levofloxacin, chlorhexidine diacetate (Fluka Chemie GmbH, Buchs, Switzerland); norfloxacin, oxacillin, penicillin, vancomycin, chloramphenicol, tetracycline, EtBr (ethidium bromide), CET (cetrimide), pentamidine isothionate salt, cetylpyridinium chloride, benzalkonium chloride, tetraphenylphosphonium bromide, chlorhexidine digluconate, dequalinium chloride, VER (verapamil) and TZ (thioridazine) (Sigma-Aldrich, St. Louis, MO, USA). Stock solutions were prepared in deionized water, with the exception of tetracycline and chloramphenicol, prepared in 95 % ethanol. All solutions were prepared on the day of the experiment and kept protected from the light.

#### Determination of MICs

Cultures were grown in Mueller-Hinton broth (Oxoid Ltd., Basingstoke, UK) at 37 °C. MICs of antibiotics were determined by the two-fold broth microdilution method and evaluated according to the CLSI breakpoints [27]. MICs of biocides and dyes were also determined using the two-fold broth microdilution method during an18 h incubation period at 37 °C.

#### MICs in the presence of efflux inhibitors

The inhibitory effect of the efflux inhibitors TZ and VER on the susceptibility of the EP substrates CIP, CET and EtBr was evaluated by the two-fold microdilution method in the same conditions but in medium containing additionally a sub-inhibitory concentration of the efflux inhibitor, guaranteeing no effect on cell viability, as follows: 12.5 mg/L TZ and 200 mg/L VER [14]. After an 18 h incubation period at 37 °C, the presence of bacterial growth was evaluated visually and the lowest concentration of antimicrobial that presented no visible growth was registered as the MIC. All assays were performed in triplicate.

#### Exposure procedure

Strains ATCC25923, SM2 and SM50 were serially exposed during 20 days to constant concentrations equivalent to half their MIC or the MIC value of the EP substrates EtBr, CIP or CET (Additional file 1: Figure S1). Cultures were grown overnight in tryptone soy broth (P0) (TSB, Oxoid) and then diluted 100-fold in TSB (control) or in TSB supplemented with the EP substrates, considering the following MICs: EtBr, 6.25 mg/L (ATCC25923) and 8 mg/L (SM2, SM50); CIP, 0.25 mg/L (ATCC25923), 32 mg/L (SM2) and 64 mg/L (SM50); CET, 2 mg/L (ATCC25923, SM2), 4 mg/L (SM50). The cultures were incubated at 37 °C with shaking and after 18 h an aliquot was diluted 100-fold in media supplemented with the same concentration of the substrate and grown in the same conditions. This procedure was repeated through 20 passages in 20 days (P20), after the first culture (P1) was obtained. The response of the strains to each EP substrate was monitored at several time points by MIC determination to EP substrates (P0 – P3, P20) and to a wide panel of antibiotics and biocides (P0, P20) in the absence and presence of efflux inhibitors (Additional file 1: Figure S1).

#### PFGE analysis of *Sma*I macrorestriction profiles

The identity of strains before (P0) and after (P20) antimicrobial exposure was verified by PFGE analysis of *Sma*I macrorestriction profiles using a previously described protocol [14, 42].

#### Assessment of EtBr efflux activity by real-time- fluorometry

This method allows the real-time fluorometric detection of EtBr efflux, a broad substrate of bacterial EPs [43]. The capacity of cells to efflux EtBr was evaluated as described previously [14]. Briefly, EtBr-loaded cells were obtained by incubation with 200 mg/L of VER (the efflux inhibitor that promotes the highest EtBr accumulation in *S. aureus*) [14] and the most suitable EtBr concentration for each culture. Efflux assays in EtBr-loaded cells were then conducted in the presence and absence of VER 200 mg/L and/or glucose at 0.4 % (see details in legend of Fig. 2). EtBr efflux was monitored in a Rotor-Gene 3000™ during a period of 10 min with detection of fluorescence at 535/585 nm each 10 seconds. For each assay, the raw data obtained was normalized against data of non-effluxing cells (cells plus 200 mg/L VER only), at each point, considering that these correspond to the maximum fluorescence values that can be achieved during the assay. The relative fluorescence thus corresponds to the ratio of fluorescence that remains per unit of time, relatively to the EtBr-loaded cells [43]. The slope (m) of each resulting EtBr efflux curve was calculated by linear regression using the values obtained during the first 1 to 2 min of the assays, as they portray the linear behavior of the EtBr extrusion from the cells reflecting the efflux responsiveness of each strain. The Relative Index of Efflux activity (RIE) was calculated using the following formula:

$$ \mathrm{R}\mathrm{I}\mathrm{E} = \frac{\left(\mathrm{R}\mathrm{F}\ \mathrm{of}\ \mathrm{cells}\ \mathrm{at}\ \mathrm{P}20\right)-\left(\mathrm{R}\mathrm{F}\ \mathrm{of}\ \mathrm{cells}\ \mathrm{at}\ \mathrm{P}0\right)}{\left(\mathrm{R}\mathrm{F}\ \mathrm{of}\ \mathrm{cells}\ \mathrm{at}\ \mathrm{P}0\right)} $$

Where RF is the relative fluorescence value at the 10^th^ minute of the efflux assay and P0 and P20 correspond to day 0 and day 20, respectively, of exposure. The RIE values allow a direct comparison of the EtBr efflux activity of each strain after exposure relatively to its initial condition, with a value of 0 corresponding to no increase in efflux activity and a value of 1 corresponding to a 100 % increase of EtBr efflux. Negative RIE values represent loss of EtBr efflux activity.

#### Gene expression analysis

The expression of the multidrug EP genes *norA*, *norB*, *norC*, *mepA*, *mdeA* and of the regulator genes *mgrA* and *mepR* was evaluated during day 1 (at mid exponential growth phase (OD_600_ of 0.6) and at the 18^th^ h of growth) and day 20 (18^th^ h of growth) of exposure (Additional file 1: Figure S1). At these time points, a 2 mL aliquot of culture was transferred to a mixture of 1:1 acetone:ethanol and immediately kept at −20 °C for RNA extraction. Total RNA was isolated by the Trizol method [44] using TRI reagent (Sigma). RNA was quantified in a NanoDrop 1000 (ThermoScientific, Madison, WI, USA) and its integrity evaluated by 1 % agarose-2.2 M formaldehyde gel electrophoresis. Quantitative RT-PCR (RT-qPCR) was performed using the QuantiTect SYBR Green RT-PCR Kit (QIAGEN, Hilden, Germany) in a Rotor-Gene 3000™ thermocycler and equivalent RNA quantities. The primers used in these assays are described in Additional file 1: Table S8. Relative gene expression was assessed by comparison of the relative quantity of each mRNA in the presence of EP substrate to the control (substrate-free condition of the corresponding time point) using the comparative threshold cycle (*C*_*T*_) method [45] and by using *gyrB* gene as reference control, with real-time analysis software. Negative and genomic DNA contamination controls were included. Genes showing increased expression of at least two-fold were considered to be overexpressed. To verify the reaction specificity, a melting curve analysis was done after each assay and the RT-qPCR products were visualized in a 2 % agarose gel electrophoresis.

#### Screening of resistance mutations in *grlA*/*gyrA* genes and promoter regions of *norA/B/C* and *mgrA*

Genomic DNA was isolated with the QIAamp DNA Mini Kit (QIAGEN), with an additional step of 30 min digestion with 0.2 mg/L of lysostaphin (Sigma) prior to extraction. Internal fragments comprising the QRDR of *grlA* (GrlA residues 14–181) and *gyrA* (GyrA residues 14–186) genes and fragments comprising the promoter regions of genes *norA/B/C* and *mgrA* were amplified using the primers described in Additional file 1: Table S8. Amplification products were sequenced in both strands using the same set of primers. Sequences were analyzed and aligned using the freeware programs BioEdit and ClustalOmega, respectively.

#### Multilocus sequence typing (MLST)

Internal fragments of the seven housekeeping genes *arcC*, *aroE*, *glpF*, *gmk*, *pta*, *tpi* and *yqiL* were amplified by PCR using the primers and conditions described previously [46, 47]. The PCR products were then sequenced on both strands using the same set of primers. The sequences were submitted to the MLST database (www.mlst.net) in order to obtain an allelic profile and sequence type (ST).

#### s*pa* typing

An internal fragment of the *spa* gene was amplified using established primers [48]. The amplified products were sequenced and the sequences submitted to the *spa*Typer free software (http://spatyper.fortinbras.us) in order to assign *spa* types.

### Availability of supporting data

The data sets supporting the results of this article are included within the article and its additional file.

## Additional file

Additional file 1:
**Supporting phenotypic and genotypic data.** Additional data for the strains in study containing MIC values of antibiotics, biocides and dyes before andafter the 20-day exposure to ethidium bromide, ciprofloxacin or cetrimide (**Tables S1** to **S3**); the effect of the efflux inhibitor verapamil on the MIC values of the three EP substrates before and after each exposure process (**Table S4**); levels of gene expression of MDR EP and regulator genes at different time points of exposure to the EP substrates (**Tables S5 to S7**); list of the primers used in this study (**Table S8**); diagram of the exposure processes to which the three strains were subjected (**Figure S1**); *SmaI* macrorestriction profiles of the parental and exposed strains (**Figure S2**). (DOCX 1137 kb)

## Abbreviations

CC: Clonal complex
CET: Cetrimide
CIP: Ciprofloxacin
CT: Threshold cycle
EI: Efflux inhibitor
EP: Efflux pump
EtBr: Ethidium bromide
MDR: Multidrug resistance
MIC: Minimum inhibitory concentration
MLST: Multilocus sequence typing
MRSA: Methicillin-resistant *Staphylococcus aureus*
MSSA: Methicillin-susceptible *Staphylococcus aureus*
P*n*: Passage at day *n*
PFGE: Pulsed-field gel electrophoresis
QRDR: Quinolone resistance-determining region
RIE: Relative index of efflux activity
RT-qPCR: Quantitative reverse transcription – polymerase chain reaction
ST: Sequence type
TSB: Tryptone soy broth
TZ: Thioridazine
VER: Verapamil
