# Supplementary material for: Impact of efflux in the development of multidrug resistance phenotypes in Staphylococcus aureus
Source: BMC Microbiol. 2015 Oct 24;15:232. doi: 10.1186/s12866-015-0572-8 (PMC4619429; doi:10.1186/s12866-015-0572-8)
Supplement: Additional file 1: — Supporting phenotypic and genotypic data. Additional data for the strains in study containing MIC values of antibiotics, biocides and dyes before andafter the 20-day exposure to ethidium bromide, ciprofloxacin or cetrimide (Tables S1 to S3); the effect of the efflux inhibitor verapamil on the MIC values of the three EP substrates before and after each exposure process (Table S4); levels of gene expression of MDR EP and regulator genes at different time points of exposure to the EP substrates (Tables S5 to S7); list of the primers used in this study (Table S8); diagram of the exposure processes to which the three strains were subjected (Figure S1); SmaI macrorestriction profiles of the parental and exposed strains (Figure S2). (DOCX 1137 kb) [file 12866_2015_572_MOESM1_ESM.docx]

Table S1. MIC values (mg/L) of antibiotics, biocides and dyes for strain ATCC25923. Data presented correspond to the MICs before and after (20 days) exposure to ethidium bromide, ciprofloxacin and cetrimide. The numbers in brackets indicate the increase of the MIC values registered at the end of each exposure process.

|  | **Original**  **MIC** |  | **MIC after exposure to:** | | | | | | | |
| --- | --- | --- | --- | --- | --- | --- | --- | --- | --- | --- |
|  |  |  | **EtBr** | |  | **CIP** | |  | **CET** | |
|  |  |  | **½ MIC** | **MIC** |  | **½ MIC** | **MIC** |  | **½ MIC** | **MIC** |
| **EtBr** | 8 |  | 32  (↑4x) | 32  (↑4x) |  | 32  (↑4x) | 32  (↑4x) |  | 8  (-) | 32  (↑4x) |
| **CIP** | 0.25 |  | 2  (↑8x) | 2  (↑8x) |  | 2  (↑8x) | 2  (↑8x) |  | 0.5  (↑2x) | 2  (↑8x) |
| **CET** | 2 |  | 4  (↑2x) | 8  (↑4x) |  | 4  (↑2x) | 4  (↑2x) |  | 4  (↑2x) | 4  (↑2x) |
| **NOR** | 0.5 |  | 8  (↑16x) | 8  (↑16x) |  | >8  (>↑16x) | >8  (>↑16x) |  | 2  (↑4x) | 8  (↑16x) |
| **LEV** | 0.25 |  | 0.5  (↑2x) | 0.5  (↑2x) |  | 0.5  (↑2x) | 1  (↑4x) |  | 0.25  (-) | 0.5  (↑2x) |
| **OXA** | 0.125 |  | 0.125  (-) | 0.125  (-) |  | 0.125  (-) | 0.125  (-) |  | 0.125  (-) | 0.125  (-) |
| **PEN** | 0.03 |  | 0.03  (-) | 0.03  (-) |  | 0.03  (-) | 0.03  (-) |  | 0.03  (-) | 0.03  (-) |
| **VAN** | 1 |  | 1  (-) | 1  (-) |  | 1  (-) | 1  (-) |  | 1  (-) | 1  (-) |
| **CHL** | 4 |  | 4  (-) | 4  (-) |  | 4  (-) | 4  (-) |  | 4  (-) | 4  (-) |
| **TET** | 0.25 |  | 0.25  (-) | 0.25  (-) |  | 0.25  (-) | 0.25  (-) |  | 0.25  (-) | 0.25  (-) |
| **PT** | 32 |  | 128  (↑4x) | 128  (↑4x) |  | 64  (↑2x) | 32  (-) |  | 32  (-) | 128  (↑4x) |
| **CPC** | 0.5 |  | 1  (↑2x) | 2  (↑4x) |  | 2  (↑4x) | 1  (↑2x) |  | 2  (↑4x) | 2  (↑4x) |
| **BAC** | 2 |  | 2  (-) | 2  (-) |  | 2  (-) | 4  (↑2x) |  | 2  (-) | 2  (-) |
| **TPP** | 16 |  | 64  (↑4x) | 64  (↑4x) |  | 64  (↑4x) | 16  (-) |  | 16  (-) | 32  (↑2x) |
| **CHX** | 1 |  | 2  (↑2x) | 2  (↑2x) |  | 2  (↑2x) | 1  (-) |  | 2  (↑2x) | 1  (-) |
| **CHXg** | 0.3 |  | 0.6  (↑2x) | 1.25  (↑4x) |  | 0.6  (↑2x) | 0.6  (↑2x) |  | 0.6  (↑2x) | 0.6  (↑2x) |
| **DQ** | 4 |  | 8  (↑2x) | 16  (↑4x) |  | 8  (↑2x) | 4  (-) |  | 4  (-) | 8  (↑2x) |

EtBr: ethidium bromide; CIP: ciprofloxacin; CET: cetrimide; NOR: norfloxacin; LEV: levofloxacin; OXA: oxacillin; PEN: penicillin; VAN: vancomycin; CHL: chloramphenicol; TET: tetracycline; PT: pentamidine; CPC: cetylpyridinium chloride; BAC: benzalkonium chloride; TPP: tetraphenylphosphonium bromide; CHX: chlorhexidine diacetate; CHXg: chlorhexidine digluconate; DQ: dequalinium chloride.

Table S2. MIC values (mg/L) of antibiotics, biocides and dyes for strain SM2. Data presented correspond to the MICs before and after (20 days) exposure to ethidium bromide, ciprofloxacin and cetrimide. The numbers in brackets indicate the increase of the MIC values registered at the end of each exposure process.

|  | **Original**  **MIC** |  | **MIC after exposure to:** | | | | | | | |
| --- | --- | --- | --- | --- | --- | --- | --- | --- | --- | --- |
|  |  |  | **EtBr** | |  | **CIP** | |  | **CET** | |
|  |  |  | **½ MIC** | **MIC** |  | **½ MIC** | **MIC** |  | **½ MIC** | **MIC** |
| **EtBr** | 8 |  | 32  (↑4x) | 64  (↑8x) |  | 32  (↑4x) | 64  (↑8x) |  | 8  (-) | 32  (↑4x) |
| **CIP** | 32 |  | 128  (↑4x) | 512 (↑16x) |  | 256  (↑8x) | 256  (↑8x) |  | 32  (-) | 128 (↑4x) |
| **CET** | 2 |  | 4  (↑2x) | 8  (↑4x) |  | 4  (↑2x) | 8  (↑4x) |  | 4  (↑2x) | 8  (↑4x) |
| **NOR** | 128 |  | 512  (↑4x) | >512  (>↑4x) |  | >512  (>↑4x) | >512  (>↑4x) |  | 128  (-) | 512  (↑4x) |
| **LEV** | 32 |  | 32  (-) | 64  (↑2x) |  | 64  (↑2x) | 64  (↑2x) |  | 16  (↓2x) | 32  (-) |
| **OXA** | 256 |  | 512  (↑2x) | 512  (↑2x) |  | 512  (↑2x) | 512  (↑2x) |  | 256  (-) | 512  (↑2x) |
| **PEN** | 128 |  | 128  (-) | 128  (-) |  | 128  (-) | 128  (-) |  | 128  (-) | 256  (↑2x) |
| **VAN** | 1 |  | 1  (-) | 1  (-) |  | 1  (-) | 1  (-) |  | 1  (-) | 1  (-) |
| **CHL** | 8 |  | 8  (-) | 8  (-) |  | 8  (-) | 8  (-) |  | 8  (-) | 8  (-) |
| **TET** | 16 |  | 16  (-) | 16  (-) |  | 16  (-) | 16  (-) |  | 16  (-) | 16  (-) |
| **PT** | 16 |  | 32  (↑2x) | 64  (↑4x) |  | 64  (↑4x) | 64  (↑4x) |  | 16  (-) | 64  (↑4x) |
| **CPC** | 0.5 |  | 2  (↑4x) | 4  (↑8x) |  | 2  (↑4x) | 4  (↑8x) |  | 0.5  (-) | 2  (↑4x) |
| **BAC** | 1 |  | 2  (↑2x) | 4  (↑4x) |  | 2  (↑2x) | 2  (↑2x) |  | 1  (-) | 2  (↑2x) |
| **TPP** | 32 |  | 64  (↑2x) | 128  (↑4x) |  | 128  (↑4x) | 128  (↑4x) |  | 32  (-) | 64  (↑2x) |
| **CHX** | 0.5 |  | 1  (↑2x) | 1  (↑2x) |  | 1  (↑2x) | 1  (↑2x) |  | 0.5  (-) | 1  (↑2x) |
| **CHXg** | 0.6 |  | 0.6  (-) | 1.25  (↑2x) |  | 1.25  (↑2x) | 1.25  (↑2x) |  | 0.6  (-) | 1.25  (↑2x) |
| **DQ** | 4 |  | 8  (↑2x) | 16  (↑4x) |  | 16  (↑4x) | 16  (↑4x) |  | 4  (-) | 16  (↑4x) |

EtBr: ethidium bromide; CIP: ciprofloxacin; CET: cetrimide; NOR: norfloxacin; LEV: levofloxacin; OXA: oxacillin; PEN: penicillin; VAN: vancomycin; CHL: chloramphenicol; TET: tetracycline; PT: pentamidine; CPC: cetylpyridinium chloride; BAC: benzalkonium chloride; TPP: tetraphenylphosphonium bromide; CHX: chlorhexidine diacetate; CHXg: chlorhexidine digluconate; DQ: dequalinium chloride.

Table S3. MIC values (mg/L) of antibiotics, biocides and dyes for strain SM50. Data presented correspond to the MICs before and after (20 days) exposure to ethidium bromide, ciprofloxacin and cetrimide. The numbers in brackets indicate the increase of the MIC values registered at the end of each exposure process.

|  | **Original**  **MIC** |  | **MIC after exposure to:** | | | | | | | |
| --- | --- | --- | --- | --- | --- | --- | --- | --- | --- | --- |
|  |  |  | **EtBr** | |  | **CIP** | |  | **CET** | |
|  |  |  | **½ MIC** | **MIC** |  | **½ MIC** | **MIC** |  | **½ MIC** | **MIC** |
| **EtBr** | 8 |  | 64  (↑8x) | 32  (↑4x) |  | 64  (↑8x) | 32  (↑4x) |  | 16  (↑2x) | --- |
| **CIP** | 64 |  | 256  (↑4x) | 256  (↑4x) |  | 512  (↑8x) | 256  (↑4x) |  | 128 (↑2x) | --- |
| **CET** | 4 |  | 8  (↑2x) | 8  (↑2x) |  | 8  (↑2x) | 8  (↑2x) |  | 4  (-) | --- |
| **NOR** | 256 |  | >512  (>↑2x) | >512  (>↑2x) |  | >512  (>↑2x) | >512  (>↑2x) |  | 256  (-) | --- |
| **LEV** | 32 |  | 64  (↑2x) | 32  (-) |  | 64  (↑2x) | 64  (↑2x) |  | 32  (-) | --- |
| **OXA** | 256 |  | 256  (-) | 256  (-) |  | 256  (-) | 256  (-) |  | 256  (-) | --- |
| **PEN** | 128 |  | 128  (-) | 128  (-) |  | 128  (-) | 256  (↑2x) |  | 128  (-) | --- |
| **VAN** | 1 |  | 1  (-) | 1  (-) |  | 1  (-) | 1  (-) |  | 1  (-) | --- |
| **CHL** | 8 |  | 8  (-) | 8  (-) |  | 8  (-) | 8  (-) |  | 8  (-) | --- |
| **TET** | 16 |  | 16  (-) | 32  (↑2x) |  | 16  (-) | 16  (-) |  | 16  (-) | --- |
| **PT** | 16 |  | 64  (↑4x) | 64  (↑4x) |  | 64  (↑4x) | 32  (↑2x) |  | 32  (↑2x) | --- |
| **CPC** | 1 |  | 2  (↑2x) | 4  (↑4x) |  | 2  (↑2x) | 4  (↑4x) |  | 1  (-) | --- |
| **BAC** | 2 |  | 4  (↑2x) | 2  (-) |  | 2  (-) | 2  (-) |  | 2  (-) | --- |
| **TPP** | 32 |  | 128  (↑4x) | 128  (↑4x) |  | 128  (↑4x) | 128  (↑4x) |  | 64  (↑2x) | --- |
| **CHX** | 1 |  | 1  (-) | 1  (-) |  | 1  (-) | 1  (-) |  | 1  (-) | --- |
| **CHXg** | 0.6 |  | 1.25  (↑2x) | 1.25  (↑2x) |  | 0.6  (-) | 1.25  (↑2x) |  | 0.6  (-) | --- |
| **DQ** | 4 |  | 16  (↑4x) | 16  (↑4x) |  | 16  (↑4x) | 16  (↑4x) |  | 8  (↑2x) | --- |

EtBr: ethidium bromide; CIP: ciprofloxacin; CET: cetrimide; NOR: norfloxacin; LEV: levofloxacin; OXA: oxacillin; PEN: penicillin; VAN: vancomycin; CHL: chloramphenicol; TET: tetracycline; PT: pentamidine; CPC: cetylpyridinium chloride; BAC: benzalkonium chloride; TPP: tetraphenylphosphonium bromide; CHX: chlorhexidine diacetate; CHXg: chlorhexidine digluconate; DQ: dequalinium chloride.

Table S4. Effect of the efflux inhibitor verapamil (VER) on the MIC values of the three EP substrates at the beginning and at the end of exposure for the strains in study.

|  | | | | | **MIC (mg/L) after exposure to:** | | | | | | | | | | | | | | | | | | | | | | | | | | | | | | | | | | | | | | | | |  |  |
| --- | --- | --- | --- | --- | --- | --- | --- | --- | --- | --- | --- | --- | --- | --- | --- | --- | --- | --- | --- | --- | --- | --- | --- | --- | --- | --- | --- | --- | --- | --- | --- | --- | --- | --- | --- | --- | --- | --- | --- | --- | --- | --- | --- | --- | --- | --- | --- |
|  | **Original MIC (mg/L)** | |  | | | **EtBr** | | | | |  | | | **CIP** | | | | | | | | | | | | | | |  | | | **CET** | | | | | | | | | | | | | | |  |
|  |  |  |  |  |  | **½ MIC** | |  | **1x MIC** | |  | | | **½ MIC** | | | | | |  | | | **1x MIC** | | | | | |  | | | **½ MIC** | | | | | |  | | | **1x MIC** | | | | | |  |
|  | **No**  **EI** | **+**  **VER** |  | | | **No EI** | **+ VER** |  | **No EI** | **+ VER** |  | | | **No EI** | | | **+ VER** | | |  | | | **No EI** | | | **+ VER** | | |  | | | **No EI** | | | **+ VER** | | |  | | | **No EI** | | | **+ VER** | | |  |
| ***ATCC25923*** | | | | | | | | | | | | | | | | | | | | | | | | | | | | | | | | | | | | | | | | | | | | | |  |  |
| **EtBr** | 8 | 2  (↓4x) |  | | | 32 | 4  (↓8x) |  | 32 | 4  (↓8x) | |  | | | 32 | | | 16  (↓2x) | | |  | | | 32 | | | 8  (↓4x) | | |  | | | 8 | | | 2  (↓4x) | | |  | | | 32 | | | 8  (↓4x) | | |
| **CIP** | 0.25 | 0.125  (↓2x) |  | | | 2 | 0.5  (↓4x) |  | 2 | 0.25  (↓8x) | |  | | | 2 | | | 1  (↓2x) | | |  | | | 2 | | | 1  (↓2x) | | |  | | | 0.5 | | | 0.25  (↓2x) | | |  | | | 2 | | | 0.5  (↓4x) | | |
| **CET** | 2 | 0.5  (↓4x) |  | | | 4 | 1  (↓4x) |  | 8 | 0.5  (↓16x) | |  | | | 4 | | | 2  (↓2x) | | |  | | | 4 | | | 2  (↓2x) | | |  | | | 4 | | | 2  (↓2x) | | |  | | | 4 | | | 2  (↓2x) | | |
| ***SM2*** | | | | | | | | | | | | | | | | | | | | | | | | | | | | | | | | | | | | | | | | | | | | | |  |  |
| **EtBr** | 8 | 2  (↓4x) | |  | | 32 | 4  (↓8x) |  | 64 | 16  (↓4x) |  | | 32 | | | 8  (↓4x) | | |  | | | 32 | | | 8  (↓4x) | | |  | | | 8 | | | 2  (↓4x) | | |  | | | 32 | | | 4  (↓8x) | | |  |  |
| **CIP** | 32 | 16  (↓4x) | |  | | 128 | 32  (↓4x) |  | 512 | 256  (↓2x) |  | | 256 | | | 64  (↓4x) | | |  | | | 256 | | | 64  (↓4x) | | |  | | | 32 | | | 32  (-) | | |  | | | 128 | | | 64  (↓2x) | | |  |  |
| **CET** | 2 | 1  (↓2x) | |  | | 4 | 1  (↓4x) |  | 16 | 8  (↓2x) |  | | 4 | | | 4  (-) | | |  | | | 8 | | | 2  (↓4x) | | |  | | | 4 | | | 2  (↓2x) | | |  | | | 8 | | | 4  (↓2x) | | |  |  |
| ***SM50*** | | | | | | | | | | | | | | | | | | | | | | | | | | | | | | | | | | | | | | | | | | | | | |  |  |
| **EtBr** | 8 | 2  (↓4x) | |  | | 64 | 8  (↓8x) |  | 32 | 8  (↓4x) |  | | 64 | | | 8  (↓8x) | | |  | | | 32 | | | 8  (↓4x) | | |  | | | 16 | | | 4  (↓4x) | | |  | | | --- | | | --- | | |  |  |
| **CIP** | 64 | 16  (↓4x) | |  | | 256 | 64  (↓4x) |  | 256 | 64  (↓4x) |  | | 512 | | | 128  (↓4x) | | |  | | | 256 | | | 64  (↓4x) | | |  | | | 128 | | | 32  (↓4x) | | |  | | | --- | | | --- | | |  |  |
| **CET** | 4 | 2  (↓2x) | |  | | 8 | 2  (↓4x) |  | 8 | 4  (↓2x) |  | | 8 | | | 4  (↓2x) | | |  | | | 8 | | | 4  (↓2x) | | |  | | | 4 | | | 2  (↓2x) | | |  | | | --- | | | --- | | |  |  |

EtBr: ethidium bromide; CIP: ciprofloxacin; CET: cetrimide, No EI: absence of efflux inhibitor; VER: verapamil; ---: no growth for strain SM50 in the presence of CET MIC. The values in brackets correspond to the decrease of the MICs in the presence of a sub-inhibitory concentration of VER relatively to the original values (absence of efflux inhibitor); decreases of ≥ 4-fold are highlighted by the shadowed values.

Table S5. Gene expression of MDR EP and regulator genes of strains in study at different time points of exposure to EtBr.

|  |  | **Gene expression during exposure to EtBr** | | | | | | | | | | | |
| --- | --- | --- | --- | --- | --- | --- | --- | --- | --- | --- | --- | --- | --- |
|  |  | **ATCC25923** | |  | | **SM2** | | |  | | **SM50** | | |
| **Time points** | | **½ MIC** | **MIC** |  | | **½ MIC** | | **MIC** |  | | **½ MIC** | | **MIC** |
| ***Early-response (day 1)*** | | | | | | | | | | | | | |
| **OD 0.6** | ***norA*** | 0.38 ± 0.33 | 0.33 ± 0.06 | | 0.49 ± 0.46 | | 0.10 ± 0.12 | | | 1.24 ± 0.24 | | 0.72 ± 0.21 | |
|  | ***norB*** | 0.24 ± 0.19 | 0.12 ± 0.12 | | 0.66 ± 0.00 | | 0.35 ± 0.12 | | | **4.57 ± 1.54** | | 0.69 ± 0.10 | |
|  | ***norC*** | 0.56 ± 0.14 | 0.44 ± 0.04 | | **2.65 ± 0.26** | | **8.24 ± 4.76** | | | 1.15 ± 0.11 | | 0.45 ± 0.07 | |
|  | ***mepA*** | 0.23 ± 0.02 | 0.20 ± 0.13 | | 0.17 ± 0.01 | | 0.05 ± 0.05 | | | 0.16 ± 0.09 | | 0.01 ± 0.01 | |
|  | ***mdeA*** | 0.42 ± 0.16 | 0.37 ± 0.09 | | 0.62 ± 0.12 | | 0.41 ± 0.08 | | | 0.67 ± 0.29 | | 0.48 ± 0.39 | |
|  | ***mgrA*** | 1.50 ± 0.71 | **2.23 ± 0.76** | | 0.59 ± 0.03 | | 0.28 ± 0.18 | | | 0.69 ± 0.26 | | 0.43 ± 0.54 | |
|  | ***mepR*** | 0.68 ± 0.03 | 1.14 ± 0.54 | | 0.77 ± 0.22 | | 1.07 ± 0.11 | | | 0.93 ± 0.00 | | 0.66 ± 0.00 | |
| **18 h** | ***norA*** | 1.26 ± 0.36 | 0.46 ± 0.16 | | **4.46 ± 0.65** | | 0.34 ± 0.28 | | | 0.54 ± 1.30 | | 0.18 ± 0.12 | |
|  | ***norB*** | 0.90 ± 0.35 | **6.06 ± 3.07** | | **1.93 ± 0.09** | | 0.66 ± 0.06 | | | 0.49 ± 0.46 | | 0.07 ± 0.04 | |
|  | ***norC*** | 0.62 ± 0.00 | 0.51 ± 0.22 | | **10.79 ± 3.13** | | 0.46 ± 0.49 | | | 0.66 ± 0.39 | | 0.64 ± 0.09 | |
|  | ***mepA*** | 1.05 ± 0.98 | 0.08 ± 0.11 | | **2.12 ± 0.71** | | 0.04 ± 0.02 | | | 0.14 ± 0.18 | | 0.02 ± 0.02 | |
|  | ***mdeA*** | 0.24 ± 0.01 | 0.31 ± 0.17 | | **8.12 ± 4.52** | | 0.30 ± 0.01 | | | 0.44 ± 0.09 | | 0.45 ± 0.23 | |
|  | ***mgrA*** | 1.54 ± 0.66 | **5.88 ± 2.25** | | 0.79 ± 0.50 | | 0.38 ± 0.33 | | | 0.58 ± 0.25 | | 0.33 ± 0.06 | |
|  | ***mepR*** | 1.61 ± 0.92 | 1.16 ± 0.50 | | **2.30 ± 0.00** | | 0.22 ± 0.16 | | | 0.54 ± 0.11 | | 0.50 ± 0.00 | |
| ***Late-response (day 20)*** | | | | | | | | | | | | | |
| **18 h** | ***norA*** | 1.38 ± 0.88 | 0.57 ± 0.52 | | 0.89 ± 0.65 | | **5.89 ± 0.86** | | | **3.62 ± 0.53** | | **7.25 ± 1.06** | |
|  | ***norB*** | **1.94 ± 0.74** | **2.77 ± 0.67** | | **2.93 ± 0.14** | | 0.75 ± 0.68 | | | **2.56 ± 0.38** | | 0.32 ± 0.02 | |
|  | ***norC*** | 1.17 ± 0.99 | 1.47 ± 1.45 | | **2.08 ± 0.30** | | 1.03 ± 1.18 | | | 1.22 ± 0.57 | | 0.54 ± 0.55 | |
|  | ***mepA*** | **30.18 ± 7.07** | **15.25 ± 4.42** | | **15.07 ± 2.94** | | 0.60 ± 0.38 | | | **5.13 ± 0.75** | | 0.06 ± 0.06 | |
|  | ***mdeA*** | 1.13 ± 0.27 | 0.62 ± 0.35 | | 0.88 ± 0.63 | | 1.00 ± 0.88 | | | 1.33 ± 0.26 | | 1.01 ± 0.56 | |
|  | ***mgrA*** | 1.40 ± 0.66 | 0.49 ± 0.63 | | **2.04 ± 0.59** | | 1.27 ± 1.23 | | | 1.47 ± 0.75 | | 1.79 ± 0.46 | |
|  | ***mepR*** | **29.00 ± 4.25** | **20.41 ± 1.00** | | **34.65 ± 5.28** | | 1.41 ± 0.47 | | | **5.91 ± 3.28** | | 0.55 ± 0.53 | |

Gene expression was measured in the presence of EtBr relatively to the drug-free condition. The results are presented as the mean and standard deviation of at least two independent assays performed with extracted total RNA. Overexpression was considered for values ≥ 2 and is represented by bold-type letters.

Table S6. Gene expression of MDR EP and regulator genes of strains in study at different time points of exposure to CIP.

|  |  | **Gene expression during exposure to CIP** | | | | | | | | | | | |
| --- | --- | --- | --- | --- | --- | --- | --- | --- | --- | --- | --- | --- | --- |
|  |  | **ATCC25923** | |  | | **SM2** | | |  | | **SM50** | | |
| **Time points** | | **½ MIC** | **MIC** |  | | **½ MIC** | | **MIC** |  | | **½ MIC** | | **MIC** |
| ***Early-response (day 1)*** | | | | | | | | | | | | | |
| **OD 0.6** | ***norA*** | 0.38 ± 0.16 | 0.23 ± 0.01 | | 0.17 ± 0.08 | | **3.06 ± 0.46** | | | **5.74 ± 3.19** | | 1.40 ± 0.29 | |
|  | ***norB*** | 0.44 ± 0.62 | 0.17 ± 0.07 | | 0.90 ± 0.04 | | 0.98 ± 0.24 | | | 0.47 ± 0.65 | | 0.16 ± 0.03 | |
|  | ***norC*** | 0.46 ± 0.58 | 0.25 ± 0.11 | | **4.45 ± 1.70** | | 0.59 ± 0.03 | | | 0.72 ± 0.73 | | 1.21 ± 0.29 | |
|  | ***mepA*** | 0.36 ± 0.49 | 0.66 ± 0.70 | | 0.30 ± 0.33 | | 0.35 ± 0.16 | | | 0.10 ± 0.36 | | 0.33 ± 0.24 | |
|  | ***mdeA*** | 0.62 ± 0.06 | 0.47 ± 0.05 | | 1.14 ± 0.38 | | 1.23 ± 0.12 | | | 1.33 ± 0.26 | | 0.77 ± 0.22 | |
|  | ***mgrA*** | **2.12 ± 1.00** | 0.88± 0.38 | | 1.55 ± 1.05 | | 0.66 ± 0.58 | | | 0.83 ± 0.74 | | **2.94 ± 0.43** | |
|  | ***mepR*** | 0.97 ± 0.05 | 0.94 ± 0.18 | | **2.56 ± 0.38** | | 1.78 ± 0.52 | | | 1.05 ± 0.64 | | 0.76 ± 0.00 | |
| **18 h** | ***norA*** | 0.23 ± 0.33 | **5.35 ± 2.29** | | 0.25 ± 0.15 | | 0.43 ± 0.47 | | | nd | | 1.31 ± 0.91 | |
|  | ***norB*** | 0.13 ± 0.19 | **6.37 ± 1.55** | | 0.22 ± 0.16 | | 0.13 ± 0.00 | | | nd | | 0.05 ± 0.03 | |
|  | ***norC*** | 0.83 ± 0.56 | **7.57 ± 3.96** | | 0.67 ± 0.13 | | 0.58 ± 0.25 | | | nd | | 0.56 ± 0.14 | |
|  | ***mepA*** | 1.35 ± 0.39 | **30.41 ± 7.30** | | 0.16 ± 0.02 | | 0.03 ± 0.01 | | | nd | | 0.01 ± 0.01 | |
|  | ***mdeA*** | 0.43 ± 0.40 | **2.45 ± 0.82** | | 0.25 ± 0.08 | | 0.15 ± 0.08 | | | nd | | **4.24 ± 0.20** | |
|  | ***mgrA*** | **3.38 ± 0.50** | **5.86 ± 0.29** | | 0.68 ± 0.46 | | 0.64 ± 0.03 | | | nd | | 0.65 ± 0.31 | |
|  | ***mepR*** | 1.42 ± 1.00 | **17.38 ± 7.43** | | 0.52 ± 0.20 | | 0.34 ± 0.05 | | | nd | | 0.76 ± 0.00 | |
| ***Late-response (day 20)*** | | | | | | | | | | | | | |
| **18 h** | ***norA*** | 0.34 ± 0.18 | 1.35 ± 1.27 | | 0.33 ± 0.19 | | **6.93 ± 2.33** | | | 0.37 ± 0.09 | | 1.29 ± 0.82 | |
|  | ***norB*** | 0.79 ± 0.30 | 0.36 ± 0.20 | | 0.94 ± 0.09 | | 1.14 ± 0.38 | | | **2.46 ± 0.00** | | **4.14 ± 0.20** | |
|  | ***norC*** | 0.95 ± 1.12 | 0.44 ± 0.38 | | **1.86 ± 0.94** | | **2.94 ± 0.43** | | | **4.45 ± 2.29** | | **7.63 ± 2.21** | |
|  | ***mepA*** | 0.12 ± 0.12 | 0.30 ± 0.04 | | 0.02 ± 0.01 | | 0.09 ± 0.00 | | | 0.21 ± 0.20 | | 1.15 ± 0.11 | |
|  | ***mdeA*** | 1.86 ± 1.11 | 0.52 ± 0.20 | | 0.97 ± 0.05 | | 0.58 ± 0.11 | | | 1.09 ± 0.61 | | 0.53 ± 0.25 | |
|  | ***mgrA*** | 1.07 ± 0.36 | 0.81 ± 0.72 | | 0.86 ± 0.65 | | 1.35 ± 0.91 | | | 1.37 ± 0.20 | | 0.55 ± 0.37 | |
|  | ***mepR*** | 0.62 ± 0.27 | 0.95 ± 0.28 | | 1.28 ± 0.66 | | 0.93 ± 0.00 | | | 0.31 ± 0.18 | | 0.66 ± 0.06 | |

Gene expression was measured in the presence of CIP relatively to the drug-free condition. The results are presented as the mean and standard deviation of at least two independent assays performed with extracted total RNA. Overexpression was considered for values ≥ 2 and is represented by bold-type letters.

Table S7. Gene expression of MDR EP and regulator genes of strains in study at different time points of exposure to CET.

|  |  | **Gene expression during exposure to CET** | | | | | | | | | |
| --- | --- | --- | --- | --- | --- | --- | --- | --- | --- | --- | --- |
|  |  | **ATCC25923** | |  | | **SM2** | | |  | | **SM50** |
| **Time points** | | **½ MIC** | **MIC** |  | | **½ MIC** | | **MIC** |  | | **½ MIC** |
| ***Early-response (day 1)*** | | | | | | | | | | | |
| **OD 0.6** | ***norA*** | 0.52 ± 0.20 | 0.75 ± 0.56 | | **4.87 ± 1.62** | | **4.35 ± 1.71** | | | **2.38 ± 0.12** | |
|  | ***norB*** | 0.02 ± 0.01 | 0.64 ± 0.09 | | 0.65 ± 0.31 | | 1.17 ± 0.65 | | | 0.25 ± 0.35 | |
|  | ***norC*** | 0.48 ± 0.32 | 0.76 ± 0.79 | | **4.63 ± 2.33** | | **4.66 ± 1.99** | | | 0.76 ± 1.05 | |
|  | ***mepA*** | 0.34 ± 0.33 | 0.49 ± 0.45 | | **10.50 ±3.53** | | **15.64 ± 10.6** | | | 0.44 ± 0.61 | |
|  | ***mdeA*** | 0.91 ± 0.13 | 0.94 ± 0.09 | | 1.19 ± 0.06 | | **1.93 ± 0.62** | | | 0.75 ± 0.25 | |
|  | ***mgrA*** | 1.29 ± 0.31 | **2.22 ± 0.11** | | 0.42 ± 0.02 | | 0.99 ± 0.90 | | | **6.00 ± 2.83** | |
|  | ***mepR*** | 0.79 ± 0.04 | **2.22 ± 0.11** | | 1.57 ± 0.60 | | **4.16 ± 0.61** | | | **3.62 ± 0.53** | |
| **18 h** | ***norA*** | 0.86 ± 1.15 | **7.83 ± 3.61** | | **4.51 ± 1.09** | | **4.44 ± 0.22** | | | 0.14 ± 0.05 | |
|  | ***norB*** | 0.26 ± 0.29 | **45.40 ± 8.77** | | **2.16 ± 0.42** | | 1.34 ± 0.75 | | | 0.19 ± 0.11 | |
|  | ***norC*** | 1.29 ± 0.31 | **39.17 ± 23.40** | | **21.97 ± 3.22** | | **3.61 ± 1.64** | | | 0.64 ± 0.83 | |
|  | ***mepA*** | 0.79 ± 0.30 | **6.37 ± 1.55** | | **6.19 ±1.80** | | **8.30 ± 4.27** | | | 0.12 ± 0.07 | |
|  | ***mdeA*** | 0.73 ± 0.38 | **5.91 ± 3.77** | | **4.44 ±0.22** | | **4.78 ± 0.70** | | | 0.17 ± 0.07 | |
|  | ***mgrA*** | **1.82 ± 0.71** | **35.93 ± 15.56** | | **2.30 ± 0.00** | | **4.55 ± 2.41** | | | 0.03 ± 0.04 | |
|  | ***mepR*** | 0.85 ± 0.12 | **24.12 ± 8.12** | | **3.22 ± 1.07** | | **4.45 ±1.70** | | | 0.19 ± 0.14 | |
| ***Late-response (day 20)*** | | | | | | | | | | | |
| **18 h** | ***norA*** | 0.57 ± 0.34 | 1.38 ± 0.88 | | 0.43 ± 0.46 | | 0.88 ± 0.63 | | | 0.66 ± 0.47 | |
|  | ***norB*** | 0.46 ± 0.50 | 0.39 ± 0.56 | | 0.27 ± 0.05 | | 0.47 ± 0.05 | | | **2.46 ± 0.00** | |
|  | ***norC*** | 0.07 ± 0.01 | 0.32 ± 0.41 | | 1.74 ± 0.30 | | **3.07 ± 0.93** | | | **3.98 ± 1.34** | |
|  | ***mepA*** | 0.30 ± 0.24 | 0.13 ± 0.18 | | 0.25 ± 0.08 | | 0.50 ± 0.44 | | | 1.33 ± 0.26 | |
|  | ***mdeA*** | 1.00 ± 1.04 | 1.10 ± 1.27 | | **3.66 ± 0.89** | | 1.01 ± 0.20 | | | 1.85 ± 1.38 | |
|  | ***mgrA*** | 0.43 ± 0.10 | 0.78 ± 0.89 | | 0.89 ± 0.26 | | 1.42 ± 0.14 | | | 1.50 ± 1.12 | |
|  | ***mepR*** | 0.94 ± 0.18 | 0.67 ± 0.13 | | 1.20 ± 0.18 | | **3.62 ± 0.98** | | | 1.39 ± 0.34 | |

Gene expression was measured in the presence of CET relatively to the drug-free condition. The results are presented as the mean and standard deviation of at least two independent assays performed with extracted total RNA. Overexpression was considered for values ≥ 2 and is represented by bold-type letters. Strain SM50 showed no growth in the presence of the CET MIC.

Table S8. List of primers used in this study.

| **Primer** | **Sequence (5’-3’)** | **Amplicon Size (bp)** | **Reference** |
| --- | --- | --- | --- |
| *For RT-qPCR experiments* | | | |
| norA_Fw | TTCACCAAGCCATCAAAAAG | 95 | [1] |
| norA_Rv | CCATAAATCCACCAATCCC |  |  |
| norB_Fw | AGCGCGTTGTCTATCTTTCC | 213 | [1] |
| norB_Rv | GCAGGTGGTCTTGCTGATAA |  |  |
| norC_Fw | AATGGGTTCTAAGCGACCAA | 216 | [1] |
| norC_Rv | ATACCTGAAGCAACGCCAAC |  |  |
| mepA_Fw | TGCTGCTGCTCTGTTCTTTA | 198 | [1] |
| mepA_Rv | GCGAAGTTTCCATAATGTGC |  |  |
| mdeA_Fw | GTTTATGCGATTCGAATGGTTGGT | 155 | [1] |
| mdeA_Rv | AATTAATGCAGCTGTTCCGATAGA |  |  |
| mgrA_Fw | GGGATGAATCTCCTGTAAACG | 131 | This study |
| mgrA_Rv | TTGATCGACTTCGGAACG |  |  |
| mepR_Fw | TCGATGCACAAGATACGAGA | 111 | This study |
| mepR_Rv | GCGATACGAGTGTTTGTTCC |  |  |
| gyrB_Fw | CGTAAATCAGCGTTAGATG | 277 | [2] |
| gyrB_Rv | TCGCTAGATCAAAGTCGCCA |  |  |
| *For mutation screening* | | | |
| GrlA_Fw | AGGTGATCGCTTTGGAAGA | 501 | This study |
| GrlA_Rv | TGGTGGTATATCTGTCGCGTA |  |  |
| GyrA_Fw | CCAGTGAAATGCGTGAATC | 514 | This study |
| GyrA_Rv | TGTGGTGGAATATTCGTTGC |  |  |
| norAp_Fw | TGTTAAGTCTTGGTCATCTGCA | 449 | This study |
| norAp_Rv | AGCAGCAACAAGTAACCCTAAA |  |  |
| mgrAp_Fw | GCGATTAACTTGTCTTTGAGC | 379 | This study |
| mgrAp_Rv | TGTCAGTAACGTGGTTTTACC |  |  |

Fw: forward; Rv: reverse; bp: base pair.

[1] Costa SS, Falcão C, Viveiros M, Machado D, Martins M, Melo-Cristino J, et al. Exploring the contribution of efflux on the resistance to fluoroquinolones in clinical isolates of *Staphylococcus aureus*. BMC Microbiol. 2011;11:e241.

[2] Pan XS, Hamlyn PJ, Talens-Visconti R, Alovero FL, Manzo RH, Fisher LM. Small-colony mutants of *Staphylococcus aureus* allow selection of gyrase-mediated resistance to dual-target fluoroquinolones. Antimicrob Agents Chemother. 2002;46:2498-506.


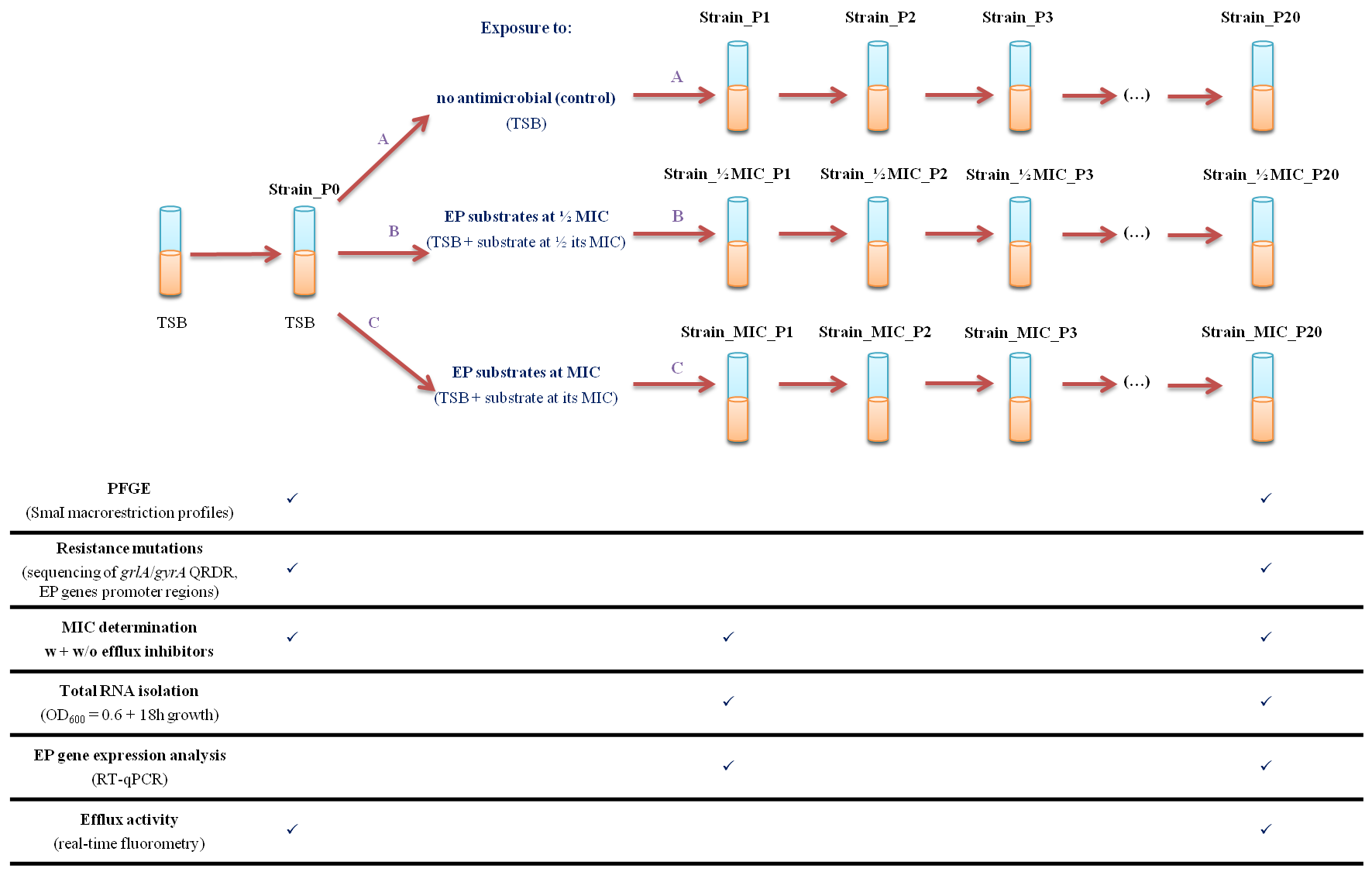


Figure S1. Diagram of the exposure processes to which the three strains in study were subjected. Each strain was subjected during 20 passages (days) after growth of the first culture (P1) to: (A) no exposure (drug-free media); (B) exposure to EP substrate at ½ its MIC; (C) exposure to EP substrate at its MIC. Growth was observed in the presence of the MIC of each compound during day 1 of most exposure regimens, albeit at different growth rates. The EP substrates used were ethidium bromide, ciprofloxacin or cetrimide. The concentration used for each exposure process considered the following MIC values: EtBr, 6.25 mg/L (ATCC25923), 8 mg/L (SM2, SM50); CIP, 0.25 mg/L (ATCC25923), 32 mg/L (SM2) and 64 mg/L (SM50); CET, 2 mg/L (ATCC25923, SM2), 4 mg/L (SM50). The several phenotypic and genotypic tests used to characterize each culture throughout the exposure process are indicated in the table. EP: efflux pump; QRDR: quinolone resistance-determining region of genes *grlA* and *gyrA*; EI: efflux inhibitor.


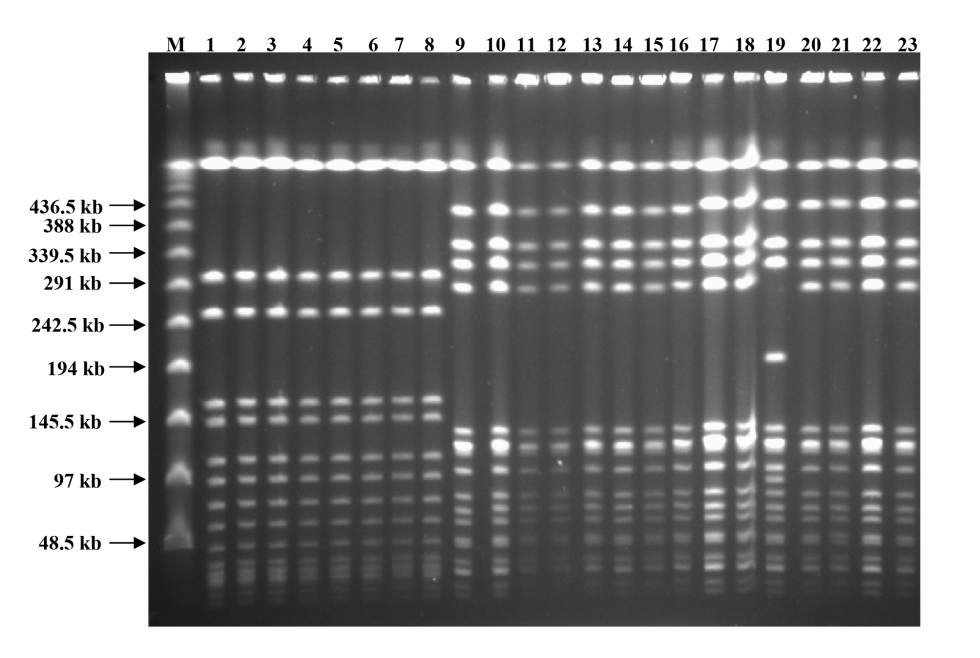


**Figure S2. *Sma*I macrorestriction profiles of the three *S. aureus* strains in study.** All strains were evaluated at the beginning and at the end of each exposure process. M – “lambda Ladder PFG marker”; 1- ATCC25923_original; 2- ATCC25923_P0 3- ATCC25923_EtBr(½MIC)_P20; 4- ATCC25923_EtBr(MIC)_P20; 5- ATCC25923_CIP(½MIC)_P20; 6- ATCC25923_CIP(MIC)_P20; 7- ATCC25923_CET(½MIC)_P20; 8- ATCC25923_CET(MIC)_P20; 9- SM2_original; 10- SM2_P0; 11- SM2_EtBr(½MIC)_P20; 12- SM2_EtBr(MIC)_P20; 13- SM2_CIP(½MIC)_P20; 14- SM2_CIP(MIC)_P20; 15- SM2_CET(½MIC)_P20; 16- SM2_CET(MIC)_P20; 17- SM50_original; 18- SM50_P0; 19- SM50_EtBr(½MIC)_P20; 20- SM50_EtBr(MIC)_P20; 21- SM50_CIP(½MIC)_P20; 22- SM50_CIP(MIC)_P20; 23- SM50_CET(½MIC)_P20.
